# Proof-of-concept PET imaging of pulmonary sarcoidosis using VAP-1-targeted radiotracer [^68^Ga]Ga-DOTA-Siglec-9

**DOI:** 10.1186/s12931-025-03455-8

**Published:** 2025-12-19

**Authors:** Prince Dadson, Heli Ylä-Outinen, Kari Kalliokoski, Terhi Tuokkola, Simona Malaspina, Mikko Koivumäki, Riikka Viitanen, Noora Rajala, Maria Silvoniemi, Tuula Tolvanen, Pirjo Nuutila, Sirpa Jalkanen, Antti Saraste, Tarja Saaresranta, Pekka Taimen, Anne Roivainen

**Affiliations:** 1 Turku PET Centre, University of Turku, Turku, Finland; 2 Turku PET Centre, Turku University Hospital, Kiinamyllynkatu 4-8, Turku, FI-20521 Finland; 3 Turku PET Centre, Åbo Akademi University, Turku, Finland; 4 Department of Pulmonary Diseases, Turku University Hospital, Turku, Finland; 5 Department of Medical Physics, Division of Medical Imaging, Turku University Hospital, Turku, Finland; 6 InFLAMES Research Flagship Center, University of Turku, Turku, Finland; 7 MediCity Research Laboratory, University of Turku, Turku, Finland; 8 Heart Center, Turku University Hospital and University of Turku, Turku, Finland; 9 Department of Pulmonary Diseases and Clinical Allergology, University of Turku, Turku, Finland; 10 Institute of Biomedicine, Department of Pathology, University of Turku, Turku University Hospital, Turku, Finland

**Keywords:** [^68^Ga]Ga-DOTA-Siglec-9, Mediastinal lymph nodes, Positron emission tomography, Pulmonary sarcoidosis, Vascular adhesion protein 1

## Abstract

**Background:**

Sarcoidosis is a multisystem granulomatous disease of unknown etiology, with pulmonary involvement being the most common and clinically significant manifestation. Vascular adhesion protein-1 (VAP-1) plays a key role in leukocyte trafficking to inflamed tissues. [⁶⁸Ga]Ga-DOTA-Siglec-9 is a novel PET radiotracer that binds to VAP-1. This proof-of-concept study aimed to evaluate the feasibility of [^68^Ga]Ga-DOTA-Siglec-9 PET/CT for imaging pulmonary sarcoidosis.

**Methods:**

Six patients with stage 2 pulmonary sarcoidosis (age 50.5 ± 13.1 years; bodyweight 84.2 ± 14.7 kg), diagnosed by clinical, radiological, and histological findings, underwent [^68^Ga]Ga-DOTA-Siglec-9 PET/CT. Control subjects included six healthy male volunteers (age 37.5 ± 10.3 years; bodyweight 80.3 ± 3.9 kg) and one female patient with lung cancer (age 77 years; bodyweight 62 kg). Tracer uptake was quantified in the lungs, mediastinal lymph nodes, and organs involved in systemic inflammation.

**Results:**

Patients with sarcoidosis showed significantly higher [^68^Ga]Ga-DOTA-Siglec-9 uptake in the lungs (SUV_mean_ 1.82 ± 0.52 vs. 0.41 ± 0.08; *P* = 0.00006) and mediastinal lymph nodes (SUV_mean_ 2.06 ± 0.46 vs. 0.89 ± 0.26; *P* = 0.0003) compared to healthy controls. Increased uptake was also observed in the liver (SUV_mean_ 1.18 ± 0.14 vs. 0.80 ± 0.10, *P* = 0.0003), spleen (SUV_mean_ 1.13 ± 0.09 vs. 0.82 ± 0.06, *P* = 0.00003), bone marrow (SUV_mean_ 0.30 ± 0.12 vs. 0.06 ± 0.05, *P* = 0.001), and bone (SUV_mean_ 0.27 ± 0.11 vs. 0.08 ± 0.04, *P =* 0.004), indicating systemic inflammation.

**Conclusions:**

This proof-of-concept study demonstrates the potential of VAP-1-targeted [^68^Ga]Ga-DOTA-Siglec-9 PET/CT for imaging pulmonary sarcoidosis and associated inflammatory activity. Further validation in larger cohorts is warranted.

**Trial registration:**

ClinicalTrials.gov, NCT03755245 (registered 27 November 2018), NCT05212103 (registered 27 January 2022).

**Graphical abstract:**

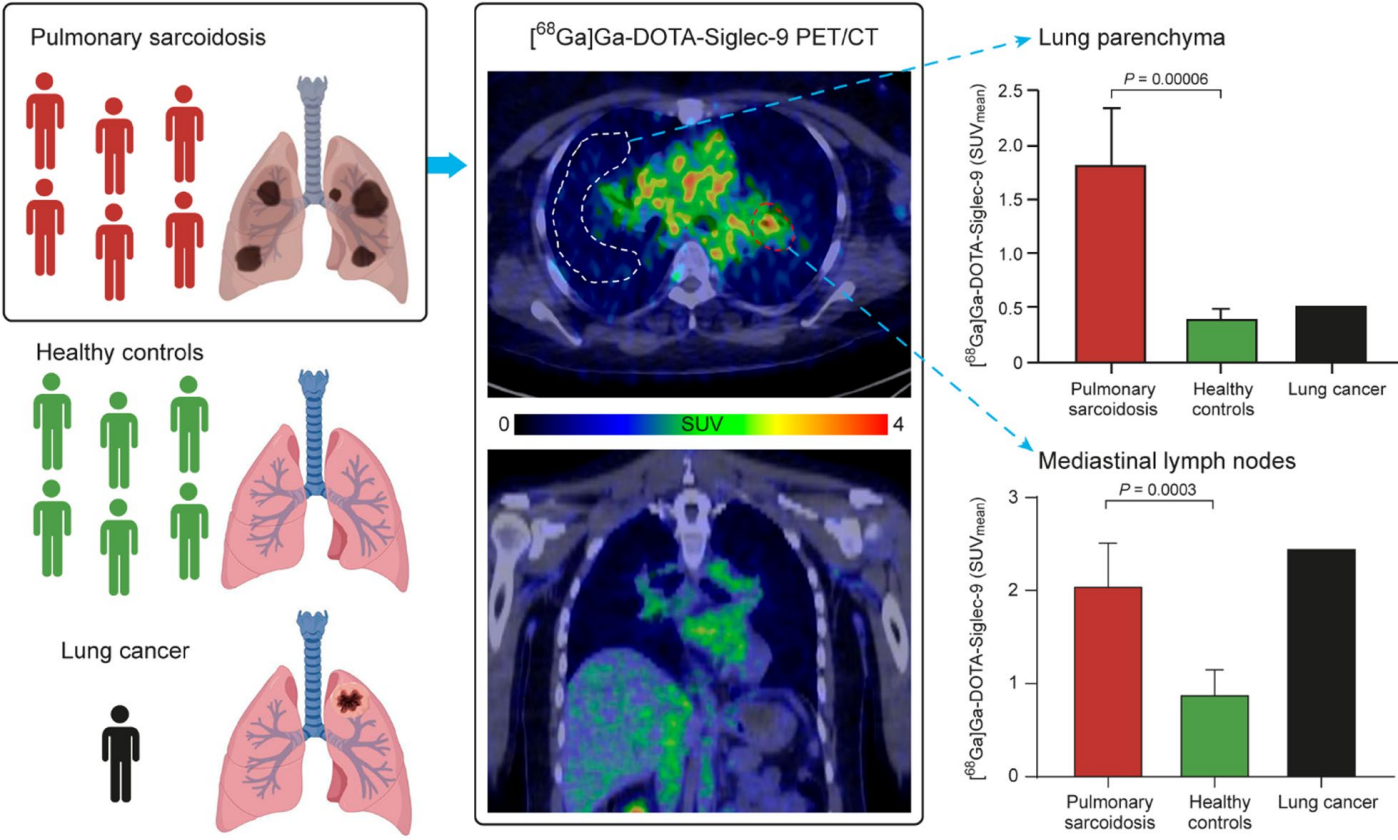

**Supplementary Information:**

The online version contains supplementary material available at 10.1186/s12931-025-03455-8.

## Introduction

Sarcoidosis is a chronic multisystem inflammatory disorder, typically characterized by the presence of noncaseating granulomas in affected organs [1]. Although its precise etiology remains unknown, sarcoidosis most commonly affects the lungs and mediastinal lymph nodes, though any organ system including heart, liver, eyes, skin, spleen, or nervous system, can be involved [2, 3]. Diagnosis is based on a combination of clinical and radiological findings, supported by histological confirmation of granulomas [4, 5]. However, in the absence of a disease-specific biomarker, noninvasive and accurate imaging modalities are urgently needed to improve diagnostic precision, especially in cases where tissue biopsy is not feasible.

2-Deoxy-2-[^18^F]fluoro-*D*-glucose ([^18^F]FDG) PET/CT is a widely used imaging modality for evaluating metabolically active granulomatous inflammation, offering high signal-to-noise ratios and good resolution [6, 7]. Despite its clinical utility, [^18^F]FDG is not specific to sarcoidosis, as it accumulates in various malignant and non-malignant inflammatory processes. This lack of specificity can complicate differential diagnosis, particularly when increased uptake is seen in lymph nodes or organs commonly involved in malignancy, such as lymphoma or metastatic disease [8, 9]. Thus, there is a pressing need for more specific molecular imaging tools that selectively target the inflammatory mechanisms unique to sarcoidosis.

Vascular adhesion protein-1 (VAP-1), also known as amine oxidase copper-containing 3 (AOC3), is a dual-function molecule involved in leukocyte trafficking. It mediates rolling, adhesion, and transendothelial migration of leukocytes during inflammation through both adhesive and enzymatic activity [10]. In resting tissues, VAP-1 resides in intracellular granules of endothelial cells, but rapidly translocates to the cell surface in response to inflammatory stimuli [11]. A soluble form of VAP-1 (sVAP-1), likely derived from the cleavage of its membrane-bound counterpart, is also detectable in circulation, with elevated levels reported in certain inflammatory diseases [12, 13].

Sialic acid-binding immunoglobulin-like lectin 9 (Siglec-9), primarily expressed on neutrophils, monocytes, macrophages, and dendritic cells [14], serves as a natural ligand for VAP-1 [15]. In response to inflammatory signals, surface expression of Siglec-9 is rapidly upregulated, making it a promising molecular probe for imaging VAP-1 activity [15]. A gallium-68-labeled peptide incorporating residues 283–297 of Siglec-9, conjugated to 1,4,7,10-tetraazacyclododecane-1,4,7,10-tetraacetic acid ([^68^Ga]Ga-DOTA-Siglec-9) has shown efficacy for PET imaging of inflammation in preclinical models of inflammatory bowel disease [16], myocarditis [17], arthritis [18], atherosclerosis [19], and acute lung injury [20]. To date, the only reported clinical use of this tracer has been in a patient with rheumatoid arthritis to image synovial inflammation [21].

In this proof-of-concept study, we investigated the feasibility of using [^68^Ga]Ga-DOTA-Siglec-9 PET/CT for VAP-1-targeted molecular imaging of pulmonary sarcoidosis and associated mediastinal lymphadenopathy.

## Materials and methods

Supplementary materials and methods are available online.

### Study population

**Fig. 1:**
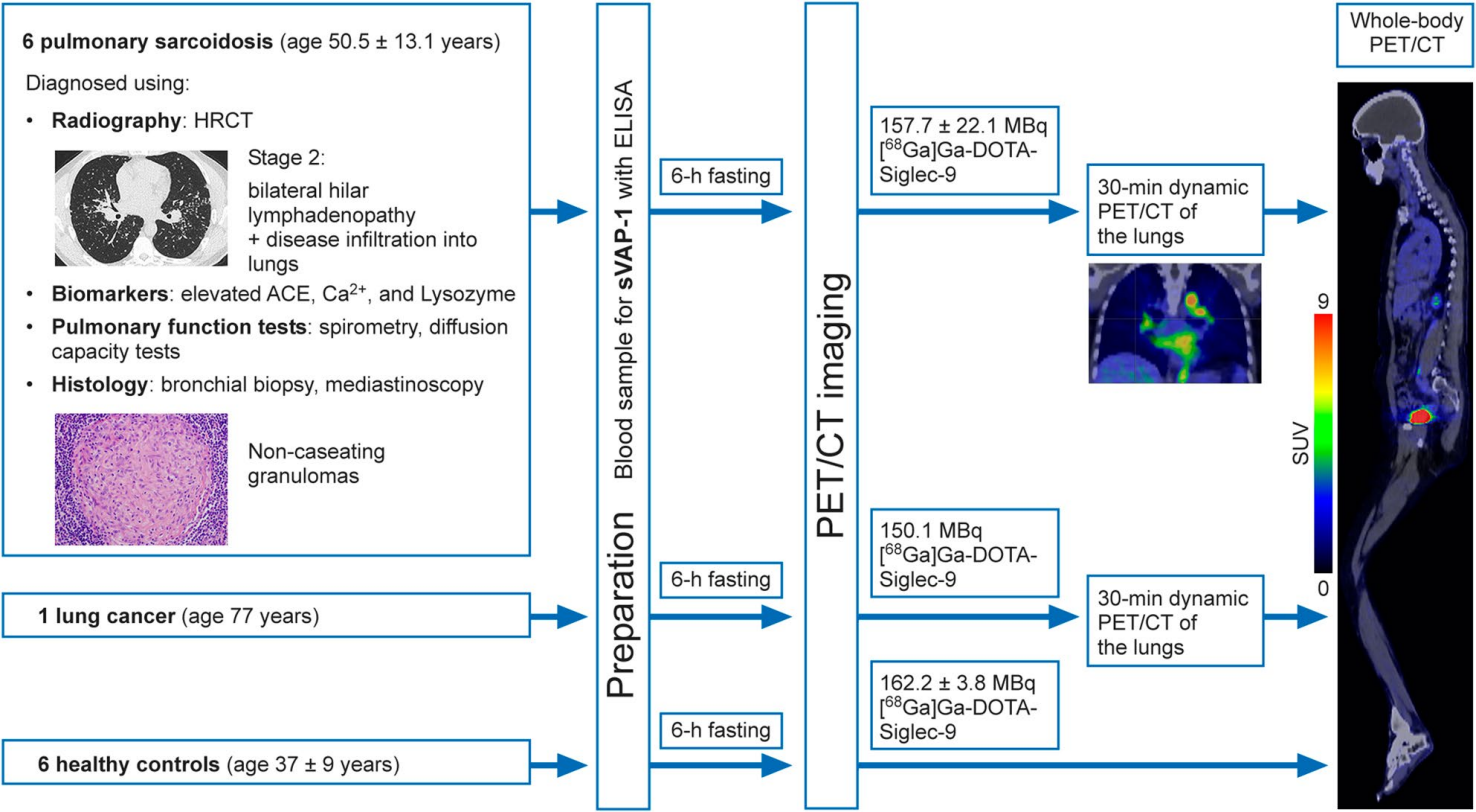
Study design

The study enrolled six patients (3 females, 3 males; age, 50.5 ± 13.1 years; body weight, 84.2 ± 14.7 kg) diagnosed with active, stage 2 pulmonary sarcoidosis, as confirmed by histology and high-resolution computed tomography (HRCT) imaging. The inclusion criteria were as follows: age 18 years or older; a diagnosis of active pulmonary sarcoidosis confirmed by histology, HRCT, and/or a serum angiotensin convertase (ACE) concentration > 60 U/L (excluding patients on ACE inhibitors); and the ability and willingness to provide written informed consent and comply with the study protocol. Exclusion criteria included diagnosis of uncontrolled type 2 diabetes, and evidence of significant uncontrolled concomitant diseases such as neurological, renal, hepatic, endocrine, or gastrointestinal disorders. Six healthy male volunteers (age 37 ± 9 years; bodyweight 80 ± 4 kg) were included as controls [21], having been recruited through local advertisements and screened at the study center. The absence of significant medical, neurological, or psychiatric history, as well as alcohol or drug abuse, was confirmed through questionnaires. Additionally, each subject underwent a medical history review, routine blood tests, electrocardiography, and physical examination. A 77-year-old female patient (weight 62 kg) with adenocarcinoma in the upper lobe of the left lung was included as a diseased control. The study design is shown in Fig. 1.

The study was approved by the joint Ethics Committee of the University of Turku and Turku University Hospital (ETMK 35/1800/2018), and by the Finnish Medicines Agency (EudraCT 2018-001002-29). Each subject provided informed consent before entering the study. The study was registered as a clinical trial (NCT03755245; registered 27 November 2018). A lung cancer patient participated in another clinical trial NCT05212103 (registered 27 January 2022; ethical permission ETMK 101/2021), but dropped out after the first scan.

### Radiopharmaceuticals

All PET radiopharmaceuticals used in the study were prepared according to Good Manufacturing Practices guidelines at the Radiopharmaceutical Laboratory of Turku PET Centre under Turku University Hospital Pharmacy control. The [^68^Ga]Ga-DOTA-Siglec-9 tracer was synthesized using an automated module [22]. The [^18^F]FDG tracer was synthesized using a FASTlab synthesizer [23].

### PET/CT scanners and radiation exposure

Imaging was conducted using the Discovery 690 and Discovery MI PET/CT systems (General Electric Medical Systems). The effective radiation dose for a 140 MBq injection of [^68^Ga]Ga-DOTA-Siglec-9 was estimated to be 3.08 mSv (0.022 mSv/MBq) [21], and that for a 200 MBq dose of [^18^F]FDG was estimated to be 3.8 mSv (0.019 mSv/MBq) [24]. In the [^18^F]FDG PET/CT study, the total radiation dose ranged from 8.0 to 12.0 mSv. In the [^68^Ga]Ga-DOTA-Siglec-9 PET/CT study, the total radiation dose ranged from 7.6 to 11.3 mSv. Imaging of a lung cancer patient was performed using Vision Quadra (Siemens Healthineers).

### PET/CT protocol

Before PET/CT, the patients underwent screening procedures and a physical examination. Females of childbearing potential had a urine pregnancy test at the time of screening and on the day of, or within 24 h prior to, the scheduled PET/CT scans. The PET/CT studies were conducted after a minimum fasting period of 6 h. A catheter was inserted into an antecubital vein for tracer injection, and another was inserted into the opposite radial vein for blood sampling to assess tracer stability and the plasma concentration of radioactivity. The subjects were positioned supine, with their arms at their sides. [^68^Ga]Ga-DOTA-Siglec-9 PET/CT and [^18^F]FDG PET/CT scans were performed on consecutive days. A low-dose whole-body CT scan (100 kV, 10−20 mA, noise index 170) was performed for attenuation correction and anatomical reference.

Sarcoidosis patients were injected intravenously with 157.7 ± 22.1 MBq of [^68^Ga]Ga-DOTA-Siglec-9 as a bolus, followed immediately by dynamic PET acquisition of the lung area over 30 min. Thereafter, a whole-body PET scan was performed from head to toe, which required approximately 12 bed positions, with a 2-min acquisition time per position. Blood samples were collected post-injection to analyze [^68^Ga]Ga-DOTA-Siglec-9 stability and the radioactivity concentration. For [^18^F]FDG PET/CT, patients were injected with 218.7 ± 59.1 MBq of the tracer, and a whole-body scan was performed 60 min later, as described above. [¹⁸F]FDG PET/CT was not performed on healthy control subjects.

Healthy control subjects received a bolus of [^68^Ga]Ga-DOTA-Siglec-9 (162.2 ± 3.8 MBq) intravenously, followed by semi-dynamic whole-body PET/CT scans as previously described [21].

The injected dose of [^68^Ga]Ga-DOTA-Siglec-9 for the diseased control subject was 150.1 MBq. After 30-min dynamic PET of the lung area, a whole-body scan was performed.

Dynamic [^68^Ga]Ga-DOTA-Siglec-9 PET images were reconstructed using VUEPoint HD (VPHD), SharpIR (General Electric), and Q.Clear, with a β value of 600. Whole-body [^68^Ga]Ga-DOTA-Siglec-9 images were reconstructed using SharpIR, as well as VUEPoint FX/Q.Clear, with a β value of 600. For the [^18^F]FDG PET scans, image reconstruction was performed using SharpIR, as well as VUEPoint FX/Q.Clear, with β value of 350.

### Quantitative analysis of PET images

**Fig. 2:**
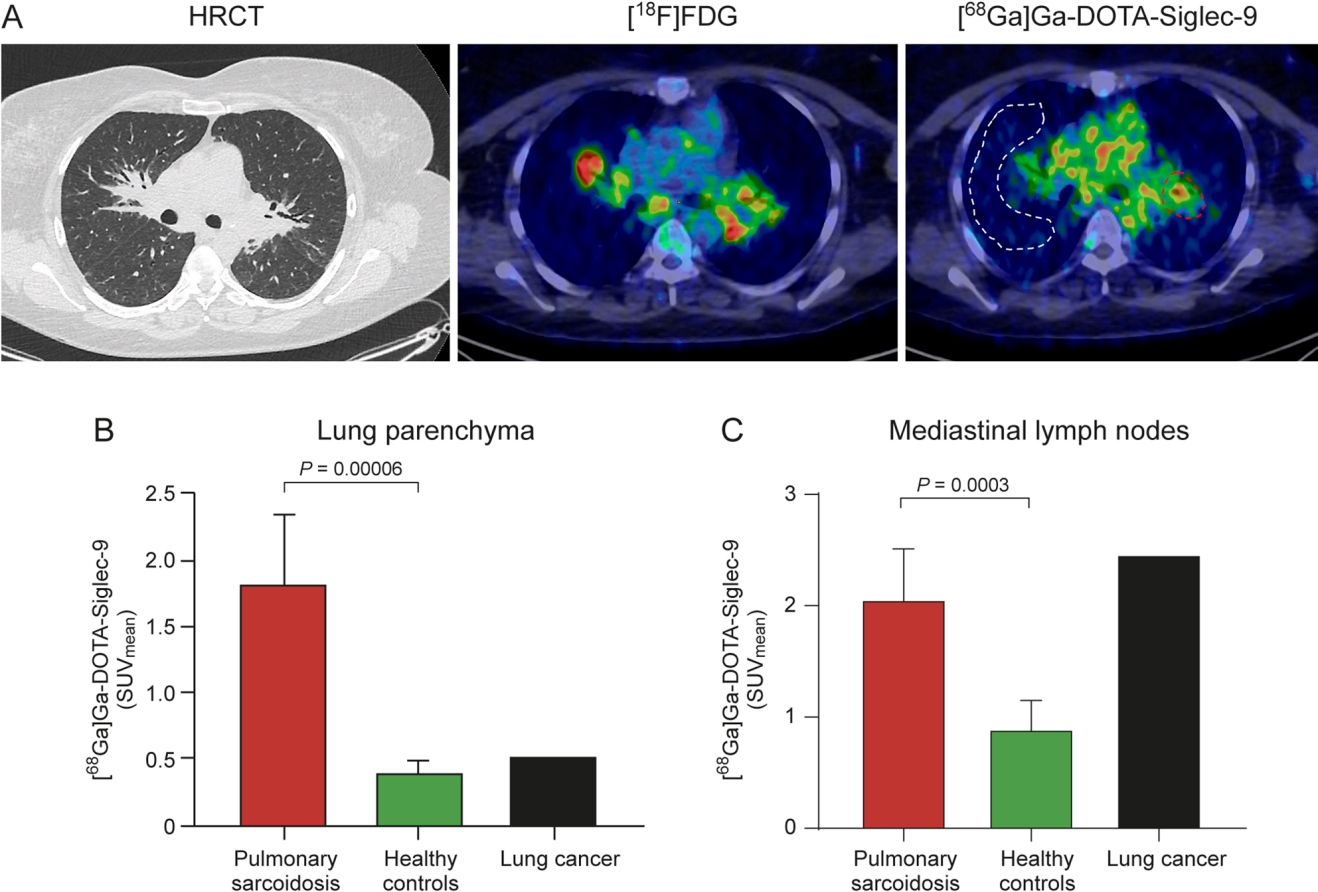
**(A**) Representative transaxial high-resolution computed tomography (HRCT), [^18^F]FDG PET/CT, and [^68^Ga]Ga-DOTA-Siglec-9 PET/CT images from a patient with stage 2 pulmonary sarcoidosis. The lung parenchymal region of interest is delineated by the dashed white line. Mediastinal lymph nodes with high uptake are delineated by red dashed lines. Quantification of [^68^Ga]Ga-DOTA-Siglec-9 PET imaging in (**B**) lung parenchyma and (**C**) mediastinal lymph nodes((

PET image analysis was performed by manually defining regions of interest (ROIs) along the anatomical boundaries of the lung parenchyma, from apex to base, on each transverse slice of the fused PET/CT images. The diaphragm, trachea, and mainstem bronchi were excluded from the ROIs. To assess lymphadenopathy, ROIs were placed on mediastinal lymph nodes at multiple stations, including the upper and lower paratracheal, subcarinal, paraesophageal, pulmonary ligament, and hilar regions (Fig. 2A). Carimas 2.10 software (Turku PET Centre) was used to define ROIs in various organs and tissues, including the cortical bone (femur), blood pool (left ventricle cavity), myocardium, kidneys, liver, lungs, skeletal muscle (triceps brachii), pancreas, femoral bone marrow, salivary glands, spleen, and urinary bladder content. The results were expressed primarily as the mean standardized uptake value (SUV_mean_), which was calculated as a ratio of tissue radioactivity concentration (Bq/mL) and given radioactivity dose (Bq) divided by subject’s body weight (kg). In addition, SUV_max_ values and target-to-blood and target-to-muscle ratios were calculated to further characterize tracer uptake and lesion-to-background contrast. Carimas software was used to visually assess myocardial uptake of [^68^Ga]Ga-DOTA-Siglec-9 on images fused with [^18^F]FDG.

### Histology and immunohistochemistry

Formalin-fixed paraffin-embedded lymph node, liver, and bronchial tissue samples were obtained from Auria Biobank (agreement #AB21-2675; Turku, Finland). For general histology, hematoxylin-eosin (HE) staining was performed on 4 μm sections. For immunohistochemistry, sections were stained with antibodies specific for VAP-1 (polyclonal VAP-1 antibody produced in rabbits against recombinant human VAP-1, dilution 1:5000, incubation overnight at + 4 °C; custom production by Harlan Sera-Lab Ltd.) or CD68 (monoclonal mouse anti-human CD68, prediluted, incubation 1.5 h at room temperature; ab845, Abcam). VAP-1 staining was performed without antigen retrieval, endogenous peroxidase blocked with Bloxall-solution (SP-6000, Vector Laboratories, Burlingame) followed by blocking with normal serum. Detection was performed with Vectastain rabbit ABC-HRP kit (PK-6101; Vector Laboratories) and 3,3’-diaminobenzidine (DAB) as a chromogen (K3468; Dako), followed by counterstaining with hematoxylin and mounting. CD68 staining was performed using fully-automated Ventana Benchmark staining system (Ventana Medical Systems), pre-treatment with Tris-based buffer (CC1 pH 8, #05424569001, Roche), and detection with Ventana ultraView Universal DAB detection kit (#05269806001, Roche). Stained sections were scanned with a digital slide scanner (Pannoramic P1000, 3DHISTECH Ltd.), and examined using Pannoramic Viewer 1.15 software (3DHISTECH Ltd.).

### sVAP-1 and other laboratory tests

Prior to tracer injection for [^68^Ga]Ga-DOTA-Siglec-9 PET/CT, serum samples were collected and the concentration of circulating sVAP-1 was measured using an in-house sandwich enzyme-linked immunosorbent assay (ELISA), as previously described [25]. In addition, blood samples were collected for hematology, serology, and clinical chemistry.

### Statistical analysis

Data are presented as the mean ± standard deviation (SD). The normality of continuous variables was assessed using the Kolmogorov-Smirnov and Shapiro-Wilk tests. Variables that deviated significantly from normality were log-transformed before statistical analyses. Independent-samples *t*-tests were used to compare normally distributed [^68^Ga]Ga-DOTA-Siglec-9 uptake values between patients with pulmonary sarcoidosis and healthy control subjects. Statistical analyses were performed using IBM SPSS version 26.0. GraphPad Prism version 8.0.2 was used to generate graphical representations of the results. A *P*-value < 0.05 was considered statistically significant.

## Results

### Characteristics of study population

The subject characteristics are summarized in Table 1. There were no significant differences between the patient and control groups regarding age (50.5 ± 13.2 vs. 37.5 ± 10.3 years; *P* = 0.088), bodyweight (84.2 ± 14.7 vs. 80.3 ± 3.9 kg; *P* = 0.561), or body mass index (BMI 26.8 ± 1.5 vs. 24.6 ± 2.6 kg/m²; *P* = 0.110). Similarly, there was no significant difference in the injected [^68^Ga]Ga-DOTA-Siglec-9 radioactivity dose (157.7 ± 22.1 vs. 162.2 ± 3.8 MBq; *P* = 0.580).

**Table 1:** Clinical and anthropometric characteristics of study subjects

	Pulmonary sarcoidosis (*n* = 6)	Healthy controls (*n* = 6)	*P* value	Lung cancer (*n* = 1)
Age (years)	50.5 ± 13.2	37.5 ± 10.3	0.088	77
Sex (female/male)	3/3	0/6	0.076	1/0
Weight (kg)	84.2 ± 14.7	80.3 ± 3.9	0.561	62
Body mass index (kg/m^2^)	26.8 ± 1.5	24.6 ± 2.6	0.110	21.0
Smoking status (smoker/non-smoker)	0/6	0/6	ND	1/0
[^68^Ga]Ga-DOTA-Siglec-9 dose (MBq)	157.7 ± 22.1	162.2 ± 3.8	0.641	150.1
Lysozyme (U/L)	22.8 ± 10.5	ND	ND	ND
Angiotensin-converting enzyme (U/L)	79.3 ± 31.9	ND	ND	ND
Calcium ions (mmol/L)	1.33 ± 0.20	ND	ND	ND
Sarcoidosis onset (months)	33.1 ± 50.8	ND	ND	ND
sVAP-1 (ng/mL)	1150.0 ± 150.2	874.0 ± 139.9	0.037	ND

The results are expressed as the mean ± SD. sVAP-1, soluble vascular adhesion protein-1

*ND* Not determined

*P* values are from the independent-samples *t*-test

The diagnosis of sarcoidosis was confirmed by the presence of granulomatous inflammation on biopsy, along with elevated levels of lysozyme (range 12−40; normal value < 12 U/L), ACE (range 49−140; normal value < 60 U/L), and calcium ions (range 1.19−1.67; normal range, 1.16−1.31 mmol/L). All patients had radiologically confirmed stage 2 pulmonary sarcoidosis. Additionally, HRCT findings in the lungs included pleural and peribronchovascular nodularity, parenchymal consolidation, ground-glass opacity, crazy paving, interlobular septal thickening, and calcification (Supplementary Table 1).

The concentration of sVAP-1 was significantly higher in subjects with pulmonary sarcoidosis than in healthy controls (1150.0 ± 150.2 vs. 874.0 ± 139.9 ng/mL; *P* = 0.037). The hematology, serology, and clinical chemistry results for the study subjects are shown in Supplementary Table 2.

### [^68^Ga]Ga-DOTA-Siglec-9 uptake in lungs and mediastinal lymph nodes

In subjects with pulmonary sarcoidosis, the [^18^F]FDG scan results revealed consistently prominent and abnormal activity in the hilar and mediastinal lymph nodes, with SUV_mean_ ranging from 4.2−12.0. Multiple hypermetabolic lymph nodes were identified in the neck, predominantly in the supraclavicular region (SUV_mean_ 3.1−6.6). Active para-aortic lymph nodes were also observed, with SUV_mean_ ranging from 9.6−19.1 (Supplementary Figs. 1–6). For descriptive purposes, the corresponding SUV_max_ values for [^18^F]FDG and [^68^Ga]Ga-DOTA-Siglec-9 in affected regions are summarized in Supplementary Table 3. It is important to note that these [^18^F]FDG images are presented for illustrative purposes only and are not intended for direct comparison. The focus of this study remains on demonstrating the imaging characteristics of [^68^Ga]Ga-DOTA-Siglec-9 PET in sarcoidosis relative to healthy controls.

**Fig. 3:**
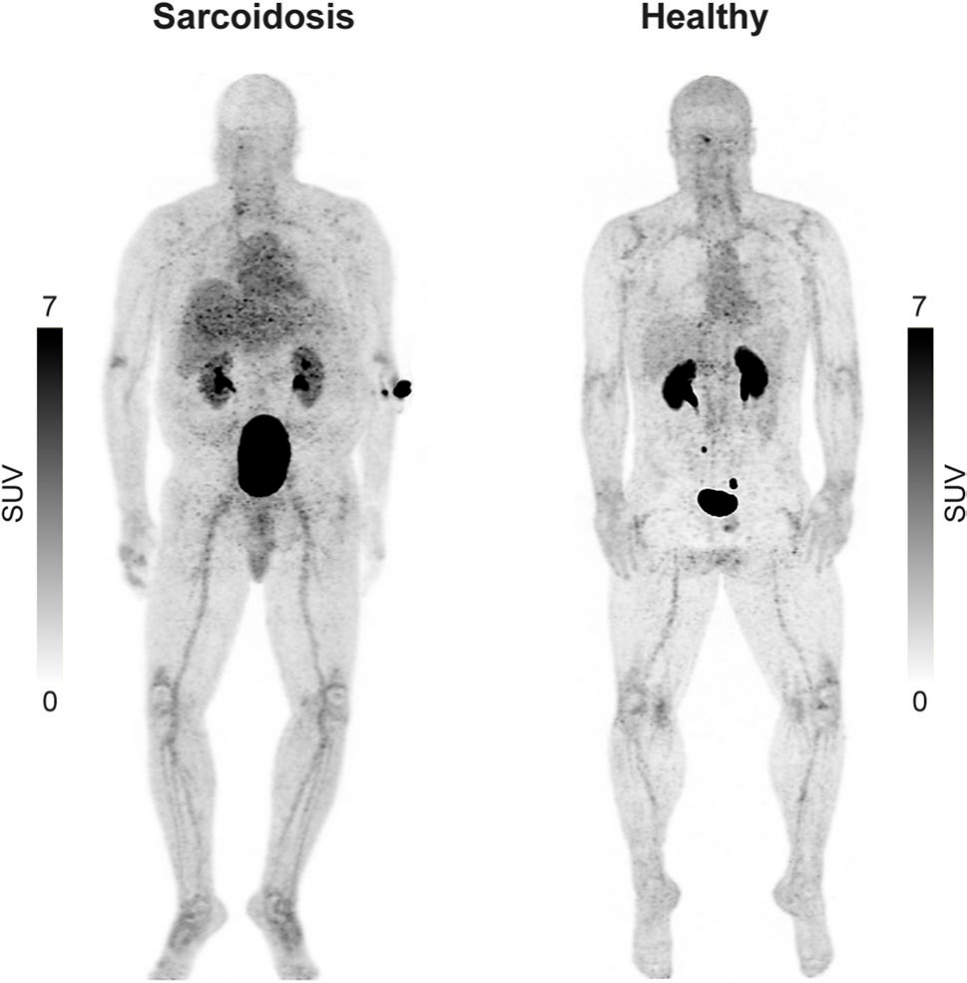
[^68^Ga]Ga-DOTA-Siglec-9 PET maximum intensity projection (MIP) images of a patient with histologically confirmed sarcoidosis and a healthy control. The patient shows increased tracer uptake in the liver and mediastinum compared with the control

We found that the uptake of [^68^Ga]Ga-DOTA-Siglec-9 in the lungs of subjects with pulmonary sarcoidosis was significantly higher than that in healthy controls (SUV_mean_ 1.82 ± 0.52 vs. 0.41 ± 0.08; *P* = 0.00006, Fig. 2B, Table 2). Additionally, uptake in mediastinal lymph nodes was significantly higher in patients than in controls (SUV_mean_ 2.06 ± 0.46 vs. 0.89 ± 0.26; *P* = 0.0003) (Fig. 2C, Table 2). In the diseased control, [^68^Ga]Ga-DOTA-Siglec-9 accumulation was observed in the lung adenocarcinoma (SUV_mean_ 1.91) and in mediastinal lymph node (SUV_mean_ 2.46). For comparison, the SUV_max_ values for [^68^Ga]Ga-DOTA-Siglec-9 uptake are summarized in Table 3. Fig. 3 shows [^68^Ga]Ga-DOTA-Siglec-9 PET images comparing a patient with sarcoidosis and a healthy control.

**Table 2:** Tissue-specific [^68^Ga]Ga-DOTA-Siglec-9 uptake as SUV_mean_

	Pulmonary sarcoidosis (*n* = 6)	Healthy controls (*n* = 6)	*P* value	Lung cancer (*n* = 1)
Blood, heart left ventricular cavity	1.48 ± 0.23	1.36 ± 0.13	0.271	2.90
Bone, cortical	0.27 ± 0.11	0.08 ± 0.04	0.004	0.59
Bone marrow	0.30 ± 0.12	0.06 ± 0.05	0.001	0.46
Kidneys	3.45 ± 0.85	6.22 ± 3.49	0.086	6.64
Liver	1.18 ± 0.14	0.80 ± 0.1	0.0003	1.81
Lung cancer lesion	ND	ND	ND	1.91
Lung parenchyma	1.82 ± 0.52	0.41 ± 0.08	0.00006	0.53
Mediastinal lymph nodes	2.06 ± 0.46	0.89 ± 0.26	0.0003	2.46
Muscle, triceps brachii	0.31 ± 0.07	0.30 ± 0.06	0.761	0.56
Myocardium	0.77 ± 0.24	0.83 ± 0.24	0.715	1.98
Pancreas	0.89 ± 0.32	0.66 ± 0.20	0.181	1.84
Parotid gland	0.61 ± 0.08	0.59 ± 0.18	0.822	1.59
Spleen	1.13 ± 0.09	0.82 ± 0.06	0.00003	1.95
Salivary gland, submandibular	0.82 ± 0.21	0.62 ± 0.17	0.093	1.95
Thymus	0.51 ± 0.16	0.91 ± 0.27	0.013	1.09
Urinary bladder content	96.52 ± 89.28	71.9 ± 45.31	0.865	33.17

The results are expressed as the mean standardized uptake values (SUV_mean_, mean ± SD)

*ND* Not determined

*P* values are from the independent-samples *t*-test

**Table 3:** Tissue-specific [^68^Ga]Ga-DOTA-Siglec-9 uptake as SUV_max_

	Pulmonary sarcoidosis (*n* = 6)	Healthy controls (*n* = 6)	*P* value	Lung cancer (*n* = 1)
Blood, heart left ventricular cavity	2.91 ± 0.86	2.17 ± 0.57	0.054	4.04
Bone, cortical	0.49 ± 0.08	0.23 ± 0.13	0.001	0.69
Bone marrow	0.58 ± 0.13	0.31 ± 0.22	0.012	0.62
Kidneys	15.56 ± 11.76	9.05 ± 2.89	0.109	6.95
Liver	2.61 ± 0.94	1.47 ± 0.46	0.012	2.30
Lung cancer lesion	ND	ND	ND	2.44
Lung parenchyma	2.01 ± 0.44	0.62 ± 0.20	0.0002	1.06
Mediastinal lymph nodes	2.49 ± 0.53	1.19 ± 0.29	0.0001	3.55
Muscle, triceps brachii	0.64 ± 0.21	0.53 ± 0.24	0.218	0.87
Myocardium	1.54 ± 0.38	1.11 ± 0.31	0.030	2.58
Pancreas	1.83 ± 0.56	1.13 ± 0.43	0.018	3.45
Parotid gland	1.09 ± 0.42	0.86 ± 0.29	0.152	2.18
Spleen	2.18 ± 0.62	1.19 ± 0.27	0.003	2.90
Salivary gland, submandibular	1.16 ± 0.48	0.94 ± 0.23	0.163	2.73
Thymus	1.10 ± 0.40	1.10 ± 0.50	0.491	1.54
Urinary bladder content	127.26 ± 140.92	80.03 ± 46.04	0.227	35.78

The results are expressed as the maximum standardized uptake values (SUV_max_, mean ± SD)

*ND* Not determined

*P* values are from the independent-samples *t*-test

**Fig. 4:**
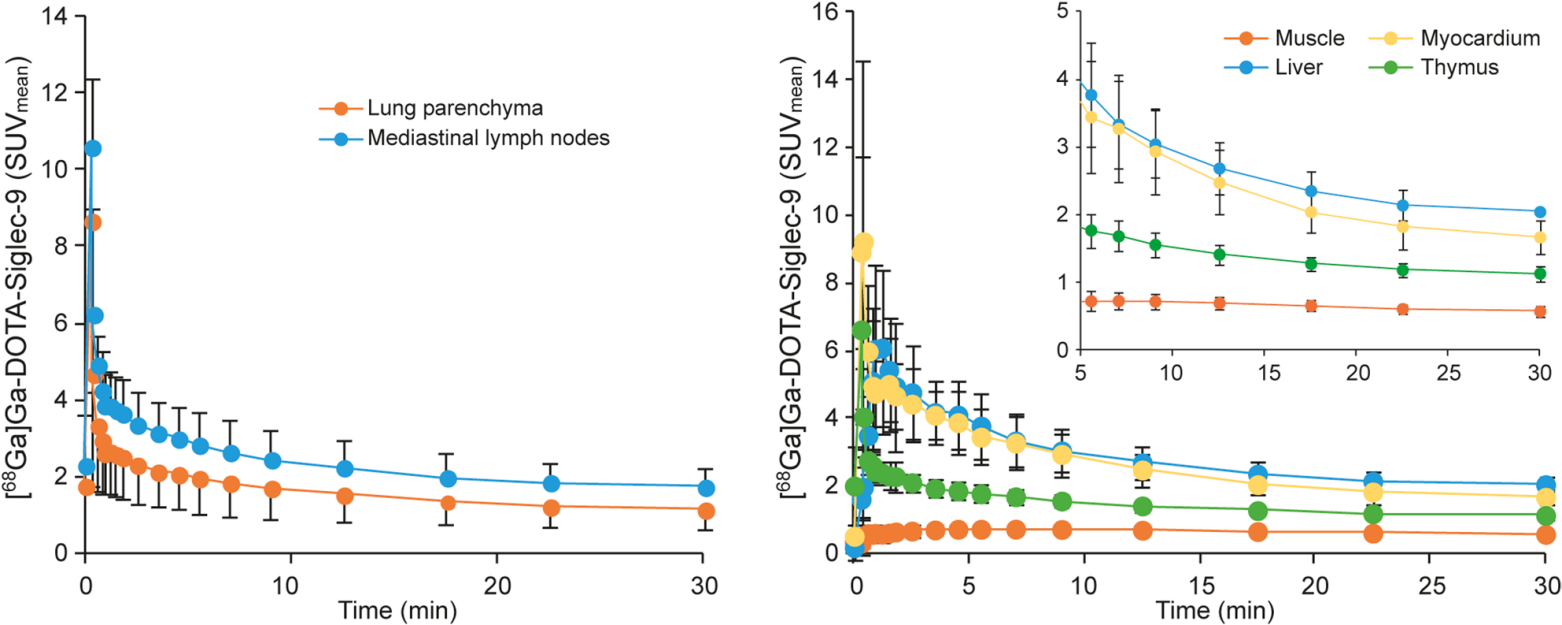
Time-activity curves for 30-min dynamic [^68^Ga]Ga-DOTA-Siglec-9 PET in the thoracic region of sarcoidosis patients show uptake in various tissues. SUV_mean_: mean standardized uptake value

The time-activity curves extracted from 30-min dynamic [^68^Ga]Ga-DOTA-Siglec-9 PET of the thoracic region confirmed higher uptake in the mediastinal lymph nodes than in the lung parenchyma of patients with sarcoidosis (Fig. 4).

### [^68^Ga]Ga-DOTA-Siglec-9 PET findings in other tissues

Uptake of [^68^Ga]Ga-DOTA-Siglec-9 in the ^cortical^bone, bone marrow, liver, and spleen, were significantly higher in patients with pulmonary sarcoidosis than in healthy controls (Table 2). Whole-body [^68^Ga]Ga-DOTA-Siglec-9 PET images (Fig. 3) highlight increased tracer uptake in the liver and mediastinum of the sarcoidosis patient compared with the healthy control*.* Conversely, the uptake in the thymus was significantly lower in patients with pulmonary sarcoidosis than in control subjects. The [^68^Ga]Ga-DOTA-Siglec-9 signal within the myocardium was homogenous and uniformly low (lower than in the blood) throughout the left ventricle in all patients (Supplementary Fig. 7). Additionally, none of the patients had a clinical history of cardiac sarcoidosis.

There was no statistically significant difference in uptake of [^68^Ga]Ga-DOTA-Siglec-9 by other organs such as the muscle, myocardium, pancreas, parotid gland, and salivary glands (Table 2), or in the plasma radioactivity concentration (Supplementary Fig. 8), between subjects with sarcoidosis and healthy controls. The time-activity curves of [^68^Ga]Ga-DOTA-Siglec-9 in sarcoidosis patients (Fig. 4) show that radioactivity concentration was highest in the liver, followed by the myocardium, thymus, and muscle, and declined to a steady level approximately 10–15 min after injection.

While the above-mentioned results are based on SUV_mean_ values, the corresponding SUV_max_ results are presented in Table 3 for comparison. Supplementary Table 3 shows that [^68^Ga]Ga-DOTA-Siglec-9 generally exhibited higher SUV_max_ than [^18^F]FDG across most tissues in patients with sarcoidosis, with significantly higher uptake observed in the blood and lung parenchyma. Tissue-to-blood and tissue-to-muscle ratios for patients with sarcoidosis and healthy controls are provided in Supplementary Tables 4 and 5, respectively.

### Histology and immunostaining

**Fig. 5:**
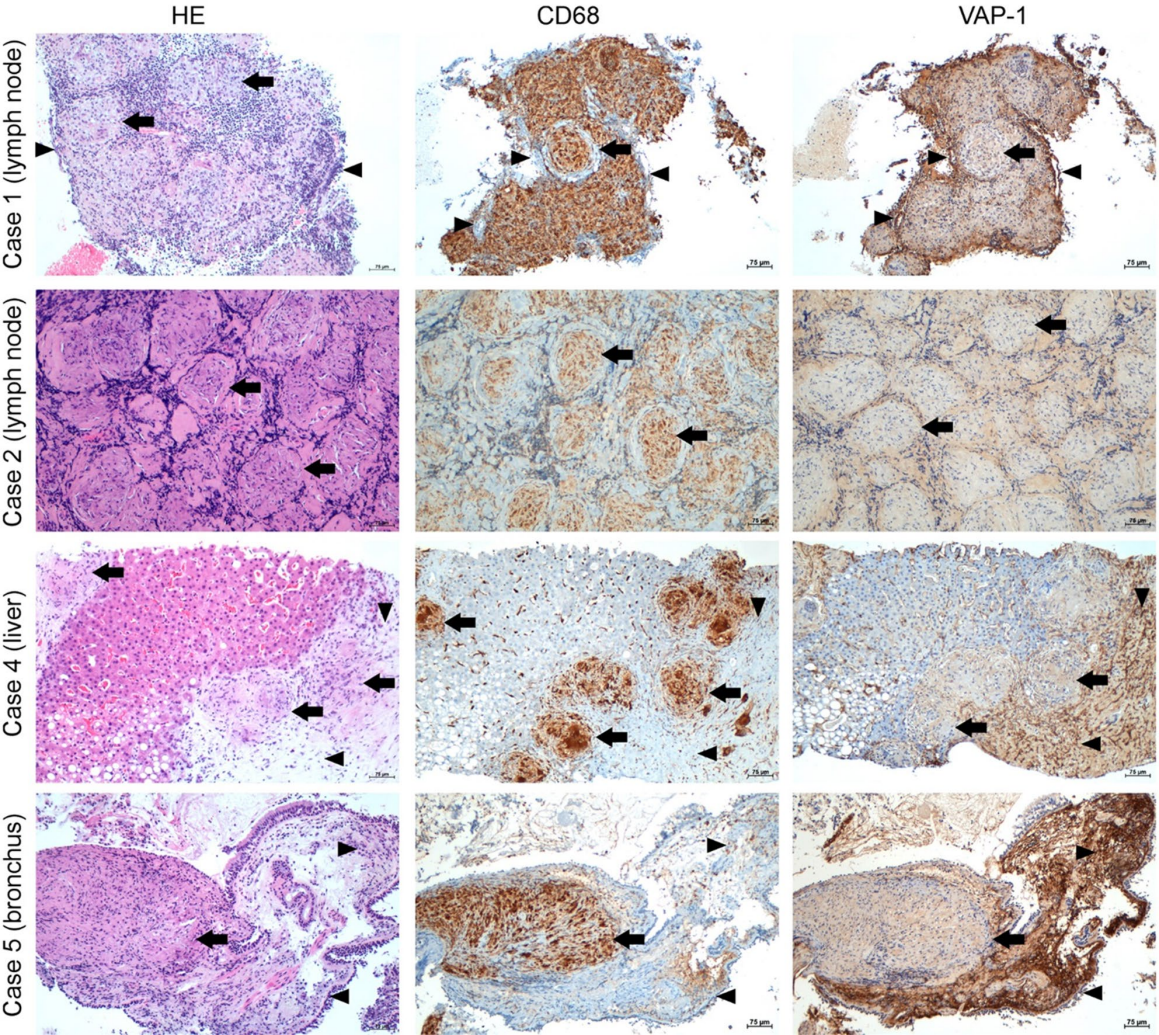
Hematoxylin-eosin (HE) staining, and immunohistochemical staining of cluster of differentiation 68 (CD68; macrophages) and vascular adhesion protein-1 (VAP-1) in lymph node (cases 1 and 2), liver (case 4), and bronchus (case 5) samples from different patients with sarcoidosis. Black arrows indicate CD68-positive sarcoid granulomas. Black arrowheads indicate VAP-1-positive endothelium and stroma

Representative images showing HE, CD68 (a marker of macrophages and histiocytes), and VAP-1 staining of lymph node, liver, and bronchus biopsies collected from study subjects diagnosed with sarcoidosis are shown in Fig. 5. In all the samples, epithelioid histiocytes (constituting sarcoid granulomas), surrounding macrophages/histiocytes, and Kupffer cells in the liver sinusoids stained positive for CD68. Endothelial cells within vascular structures of the lymph nodes were positive for VAP-1. In the liver, the sinusoidal endothelium, and especially previously injured and fibrotic areas adjacent to the granulomas, were VAP-1- positive, as were vascular endothelial cells. In addition, strong staining of the stroma beneath the basement membrane was observed.

## Discussion

This proof-of-concept study is the first to demonstrate the use of [^68^Ga]Ga-DOTA-Siglec-9 PET/CT for evaluating pulmonary sarcoidosis and associated mediastinal lymphadenopathy. Our findings reveal significantly higher tracer uptake in the lung parenchyma and mediastinal lymph nodes of patients with pulmonary sarcoidosis compared with healthy controls, indicating the potential utility of this VAP-1-targeted imaging approach for detecting active granulomatous inflammation.

Histologically, sarcoidosis is characterized by well-defined, non-necrotizing granulomas composed of epithelioid histiocytes, typically following a lymphangitic distribution [26, 27]. Although [^18^F]FDG PET/CT is widely used to evaluate sarcoidosis and other leukocyte-mediated disorders [28–32], its clinical utility is limited by non-specific uptake in any metabolically active tissue [8, 9]. This hampers differentiation between inflammatory and malignant lesions, such as lymphoma or metastatic disease. In contrast, [^68^Ga]Ga-DOTA-Siglec-9 targets VAP-1, a molecule specifically upregulated in inflamed endothelium, potentially offering greater specificity for imaging of inflammatory lesions in sarcoidosis. Our findings suggest this approach may enhance diagnostic accuracy and provide a more targeted method for monitoring disease activity.

Increased uptake of [^68^Ga]Ga-DOTA-Siglec-9 in the lungs and mediastinal lymph nodes of patients with sarcoidosis aligns with prior observations from a porcine model of acute lung injury, which also showed tracer accumulation in inflamed pulmonary tissue [33]. Similarly, VAP-1-targeted imaging has demonstrated utility in preclinical models of autoimmune myocarditis [17], where immunohistochemical studies confirmed VAP-1 expression in inflamed cardiac tissue, highlighting potential future applications in cardiac sarcoidosis.

In our study, myocardial uptake of [^68^Ga]Ga-DOTA-Siglec-9 was low in all participants, consistent with the absence of known cardiac involvement. In contrast, increased tracer uptake in the liver, spleen, bone marrow, and bone among patients with sarcoidosis suggests systemic inflammation, extending the relevance of VAP-1-targeted imaging beyond localized pulmonary findings.

[^68^Ga]Ga-DOTA-Siglec-9 was developed as a specific ligand for VAP-1, a copper-containing semicarbazide-sensitive amine oxidase (AOC3) that mediates leukocyte adhesion transmigration at sites of inflammation [34, 37]. While VAP-1 is predominantly expressed on vascular endothelial cells [34], it is also present in smooth muscle cells [38], adipocytes [39], and constitutively in the endothelial lining of organs such as the liver, kidney, and peripheral lymph nodes [34]. In our study, patients with sarcoidosis exhibited elevated serum levels sVAP-1 compared to healthy controls. Increased sVAP-1 levels have been observed in several chronic inflammatory conditions, including psoriasis, atopic eczema, chronic kidney disease, inflammatory liver disease, and multiple sclerosis [13, 40–42], though not uniformly across all inflammatory disease. Notably, sVAP-1 levels appear to vary with age and sex, with slightly elevated levels observed in younger women but no significant sex-based differences in older adults [43]. These findings support the relevance of VAP-1 both as an imaging target and potential biomarker for inflammatory activity in sarcoidosis.

Interestingly, in the diseased control with lung adenocarcinoma, [^68^Ga]Ga-DOTA-Siglec-9 uptake in the tumor and mediastinal lymph nodes was higher than the average uptake observed in patients with sarcoidosis. While VAP-1-targeted imaging shows promise for detecting inflammatory activity in sarcoidosis, it is important to note that VAP-1 is not exclusively expressed in this condition. Previous studies, including Viitanen et al. [44] have demonstrated upregulation of VAP-1 in certain malignancies such as melanoma, and our current study also observed elevated VAP-1 expression in lung adenocarcinoma. Therefore, the specificity of VAP-1-targeted imaging for sarcoidosis may be limited in contexts where malignant or other inflammatory conditions are present. Furthermore, compared to [^18^F]FDG PET, VAP-1-targeted signals were generally lower and less sensitive, highlighting that while this imaging approach provides complementary mechanistic information about inflammation, it may not detect all disease activity. Future studies are warranted to better define the sensitivity and specificity of VAP-1-targeted molecular imaging and to establish its potential role in differentiating sarcoidosis from malignant or other inflammatory conditions.

### Study limitations

This study has several limitations. Most notably, the sample size was small, and the findings must be considered preliminary. Longitudinal studies with larger and more diverse populations are needed to validate the diagnostic and prognostic value of [⁶⁸Ga]Ga-DOTA-Siglec-9 PET/CT, particularly in monitoring disease progression and treatment response. Second, comparison was limited to healthy controls and a single diseased control. Future studies should include a broader spectrum of control groups, including patients with other inflammatory and neoplastic lung diseases, to better assess the specificity and potential clinical utility of this imaging approach. Finally, the study population lacked ethnic diversity. Sarcoidosis has well-documented variations in presentation and severity across ethnic groups. For example, African American patients are more likely to experience severe pulmonary involvement, multiorgan disease, and worse outcomes—including higher hospitalization and mortality rates—than white patients [45]. These demographic factors should be considered in future research to improve generalizability and clinical relevance.

We chose SUV_mean_ to reflect the average tracer uptake across the ROIs, minimizing the influence of single-voxel noise or extreme values. We acknowledge that SUV_mean_ can be affected by ROI definition and delineation methods, which may introduce variability. Including SUV_max_ alongside SUV_mean_ provides a more comprehensive assessment and helps account for potential methodological variability.

## Conclusion

This proof-of-concept study demonstrates that VAP-1-targeted [^68^Ga]Ga-DOTA-Siglec-9 PET/CT imaging can detect inflammatory changes in pulmonary sarcoidosis and mediastinal lymphadenopathy with high contrast relative to healthy controls. The findings suggest a potential role for this tracer in noninvasive diagnosis and disease monitoring. In addition, our results contribute to the growing understanding of VAP-1’s role in sarcoidosis pathophysiology and support further development of VAP-1-targeted molecular imaging in inflammatory lung diseases.

## Supplementary Information

Supplementary Material 1.

## Data Availability

The original data of the work can be obtained from Prof. Anne Roivainen upon rational request.

## Abbreviations

[^18^F]FDG: 2-deoxy-2-[18F]fluoro-D-glucose
[^68^Ga]Ga-DOTA-Siglec-9: Gallium-68-labeled peptide incorporating residues 283–297 of Siglec-9, conjugated to 1,4,7,10-tetraazacyclododecane-1,4,7,10-tetraacetic acid
ACE: Angiotensin convertase
AOC3: Amine oxidase copper-containing 3
BMI: Body mass index
CT: Computed tomography
DAB: 3,3’-diaminobenzidine
DOTA: 1,4,7,10-tetraazacyclododecane-1,4,7,10-tetraacetic acid
ELISA: Enzyme-linked immunosorbent assay
HE: Hematoxylin-eosin
HRCT: High-resolution computed tomography
PET: Positron emission tomography
ROI: Region of interest
SD: Standard deviation
Siglec-9: Sialic acid-binding immunoglobulin-like lectin 9
SUV: Standardized uptake value
VAP-1: Vascular adhesion protein 1
sVAP-1: Soluble VAP-1

## References

[1] Jain R, Yadav D, Puranik N, Guleria R, Jin JO. Sarcoidosis: causes, diagnosis, clinical features, and treatments. J Clin Med. 2020;9:1081. 32290254 10.3390/jcm9041081 PMC7230978

[2] Statement on sarcoidosis. Joint statement of the American thoracic society (ATS), the European respiratory society (ERS) and the world association of sarcoidosis and other granulomatous disorders (WASOG) adopted by the ATS board of directors and by the ERS executive Committee, February 1999. Am J Respir Crit Care Med. 1999;160:736–55. 10430755 10.1164/ajrccm.160.2.ats4-99

[3] Grunewald J, Grutters JC, Arkema EV, Saketkoo LA, Moller DR. Müller-Quernheim J Sarcoidosis Nat Rev Dis Primer. 2019;5:45. 10.1038/s41572-019-0096-x 31273209

[4] Ungprasert P, Ryu JH, Matteson EL. Clinical manifestations, diagnosis, and treatment of sarcoidosis. Mayo Clin Proc Innov Qual Outcomes. 2019;3:358–75. 31485575 10.1016/j.mayocpiqo.2019.04.006 PMC6713839

[5] Bernardinello N, Petrarulo S, Balestro E, Cocconcelli E, Veltkamp M, Spagnolo P. Pulmonary sarcoidosis: diagnosis and differential diagnosis. Diagnostics. 2021;11:1558. 34573900 10.3390/diagnostics11091558 PMC8472810

[6] Adams H, Keijsers RG, Korenromp IH, Grutters JC. FDG PET for gauging of sarcoid disease activity. Semin Respir Crit Care Med. 2014;35:352–61. 25007087 10.1055/s-0034-1376866

[7] Keijsers RGM, Grutters JC. In which patients with sarcoidosis is FDG PET/CT indicated? J Clin Med. 2020;9:890. 32213991 10.3390/jcm9030890 PMC7141490

[8] Prabhakar HB, Rabinowitz CB, Gibbons FK, O’Donnell WJ, Shepard JA, Aquino SL. Imaging features of sarcoidosis on MDCT, FDG PET, and PET/CT. AJR Am J Roentgenol. 2008;190:S1–6. 18287458 10.2214/AJR.07.7001

[9] Bakheet SM, Powe J, Ezzat A, Rostom A. F-18-FDG uptake in tuberculosis. Clin Nucl Med. 1998;23:739–42. 9814559 10.1097/00003072-199811000-00003

[10] Merinen M, Irjala H, Salmi M, Jaakkola I, Hänninen A, Jalkanen S. Vascular adhesion protein-1 is involved in both acute and chronic inflammation in the mouse. Am J Pathol. 2005;166:793–800. 15743791 10.1016/S0002-9440(10)62300-0 PMC1602345

[11] Jaakkola K, Nikula T, Holopainen R, Vähäsilta T, Matikainen MT, Laukkanen ML, et al. In vivo detection of vascular adhesion protein-1 in experimental inflammation. Am J Pathol. 2000;157:463–71. 10934150 10.1016/S0002-9440(10)64558-0 PMC1850117

[12] Salmi M, Jalkanen S. A 90-kilodalton endothelial cell molecule mediating lymphocyte binding in humans. Science. 1992;257:1407–9. 1529341 10.1126/science.1529341

[13] Kurkijärvi R, Adams DH, Leino R, Möttönen T, Jalkanen S, Salmi M. Circulating form of human vascular adhesion protein-1 (VAP-1): increased serum levels in inflammatory liver diseases. J Immunol. 1998;161:1549–57. 9686623

[14] Zhang JQ, Nicoll G, Jones C, Crocker PR. Siglec-9, a novel Sialic acid binding member of the Immunoglobulin superfamily expressed broadly on human blood leukocytes. J Biol Chem. 2000;275:22121–26. 10801862 10.1074/jbc.M002788200

[15] Aalto K, Autio A, Kiss EA, Elima K, Nymalm Y, Veres TZ, et al. Siglec-9 is a novel leukocyte ligand for vascular adhesion protein-1 and can be used in PET imaging of inflammation and cancer. Blood. 2011;118:3725–33. 21821708 10.1182/blood-2010-09-311076 PMC3833035

[16] Bhowmik AA, Heikkilä TRH, Polari L, Virta J, Liljenbäck H, Moisio O, et al. Detection of intestinal inflammation by vascular adhesion protein-1-targeted [^68^Ga]Ga-DOTA-Siglec-9 positron emission tomography in murine models of inflammatory bowel disease. Mol Imaging Biol. 2024;26:322–33. 38110791 10.1007/s11307-023-01885-8 PMC10973022

[17] Jahandideh A, Virta J, Li XG, Liljenbäck H, Moisio O, Ponkamo J, et al. Vascular adhesion protein-1-targeted PET imaging in autoimmune myocarditis. J Nucl Cardiol. 2023;30:2760–72. 37758963 10.1007/s12350-023-03371-8 PMC10682147

[18] Siitonen R, Pietikäinen A, Liljenbäck H, Käkelä M, Söderström M, Jalkanen S, et al. Targeting of vascular adhesion protein-1 by positron emission tomography visualizes sites of inflammation in borrelia burgdorferi-infected mice. Arthritis Res Ther. 2017;19:254. 29166944 10.1186/s13075-017-1460-4 PMC5700622

[19] Silvola JMU, Virtanen H, Siitonen R, Hellberg S, Liljenbäck H, Metsälä O, et al. Leukocyte trafficking-associated vascular adhesion protein 1 is expressed and functionally active in atherosclerotic plaques. Sci Rep. 2016;6:35089. 27731409 10.1038/srep35089 PMC5059718

[20] Retamal J, Sörensen J, Lubberink M, Suarez-Sipmann F, Batista Borges J, Feinstein R, et al. Feasibility of ^68^Ga-labeled Siglec-9 peptide for the imaging of acute lung inflammation: a pilot study in a Porcine model of acute respiratory distress syndrome. Am J Nucl Med Mol Imaging. 2016;6:18–31. 27069763 PMC4749502

[21] Viitanen R, Moisio O, Lankinen P, Li X-G, Koivumäki M, Suilamo S, et al. First-in-humans study of ^68^Ga-DOTA-Siglec-9, a PET ligand targeting vascular adhesion protein 1. J Nucl Med. 2021;62:577–83. 32817143 10.2967/jnumed.120.250696 PMC8049366

[22] Käkelä M, Luoto P, Viljanen T, Virtanen H, Liljenbäck H, Jalkanen S, et al. Adventures in radiosynthesis of clinical grade [^68^Ga]Ga-DOTA-Siglec-9. RSC Adv. 2018;8:8051–6. 35542034 10.1039/c7ra12423f PMC9078465

[23] Long JZ, Jacobson MS, Hung JC. Comparison of fastlab ^18^F-FDG production using phosphate and citrate buffer cassettes. J Nucl Med Technol. 2013;41:32–4. 23318199 10.2967/jnmt.112.112649

[24] Addendum 1 to ICRP Publication 128: Radiation dose to patients from radiopharmaceuticals: a compendium of current information related to frequently used substances [Ann. ICRP 44(2S), 2015]. Ann ICRP. 2020;146645320936035. https://pubmed.ncbi.nlm.nih.gov/32663069/ . 10.1177/0146645320936035 32663069

[25] Aalto K, Havulinna AS, Jalkanen S, Salomaa V, Salmi M. Soluble vascular adhesion protein-1 predicts incident major adverse cardiovascular events and improves reclassification in a Finnish prospective cohort study. Circ Cardiovasc Genet. 2014;7:529–35. 24850810 10.1161/CIRCGENETICS.113.000543

[26] Ohshimo S, Guzman J, Costabel U, Bonella F. Differential diagnosis of granulomatous lung disease: clues and pitfalls. Eur Respir Rev. 2017;26:170012. 28794143 10.1183/16000617.0012-2017 PMC9488688

[27] Crouser ED, Maier LA, Wilson KC, Bonham CA, Morgenthau AS, Patterson KC, et al. Diagnosis and detection of Sarcoidosis. An official American thoracic society clinical practice guideline. Am J Respir Crit Care Med. 2020;201:e26–51. 32293205 10.1164/rccm.202002-0251ST PMC7159433

[28] Tana C, Donatiello I, Caputo A, Tana M, Naccarelli T, Mantini C, et al. Clinical features, histopathology and differential diagnosis of sarcoidosis. Cells. 2021;11:59. 35011621 10.3390/cells11010059 PMC8750978

[29] Mochizuki T, Tsukamoto E, Kuge Y, Kanegae K, Zhao S, Hikosaka K, et al. FDG uptake and glucose transporter subtype expressions in experimental tumor and inflammation models. J Nucl Med. 2001;42:1551–5. 11585872

[30] Israel-Biet D, Valeyre D. Diagnosis of pulmonary sarcoidosis. Curr Opin Pulm Med. 2013;19:510–5. 23880701 10.1097/MCP.0b013e3283645950

[31] Tana M, di Carlo S, Romano M, Alessandri M, Schiavone C, Montagnani A. FDG-PET/CT assessment of pulmonary sarcoidosis: A guide for internists. Curr Med Imaging Rev. 2019;15:21–5. 31964323 10.2174/1573405614666180528101755

[32] Maccarone MT. FDG-PET scan in sarcoidosis: clinical and imaging indications. Curr Med Imaging Rev. 2019;15:4–9. 31964321 10.2174/1573405614666180626120832

[33] Demaria L, Borie R, Benali K, Piekarski E, Goossens J, Palazzo E, et al. ^18^F-FDG PET/CT in bone sarcoidosis: an observational study. Clin Rheumatol. 2020;39:2727–34. 32198555 10.1007/s10067-020-05022-6

[34] Salmi M, Kalimo K, Jalkanen S. Induction and function of vascular adhesion protein-1 at sites of inflammation. J Exp Med. 1993;178:2255–60. 8245796 10.1084/jem.178.6.2255 PMC2191278

[35] Salmi M, Jalkanen S. Vascular adhesion protein-1: a cell surface amine oxidase in translation. Antioxid Redox Signal. 2019;30:314–32. 29065711 10.1089/ars.2017.7418 PMC6306676

[36] Salmi M, Rajala P, Jalkanen S. Homing of mucosal leukocytes to joints. Distinct endothelial ligands in synovium mediate leukocyte-subtype specific adhesion. J Clin Invest. 1997;99:2165–72. 9151788 10.1172/JCI119389 PMC508046

[37] Jaakkola K, Jalkanen S, Kaunismäki K, Vänttinen E, Saukko P, Alanen K, et al. Vascular adhesion protein-1, intercellular adhesion molecule-1 and P-selectin mediate leukocyte binding to ischemic heart in humans. J Am Coll Cardiol. 2000;36:122–9. 10898423 10.1016/s0735-1097(00)00706-3

[38] Jaakkola K, Kaunismäki K, Tohka S, Yegutkin G, Vänttinen E, Havia T, et al. Human vascular adhesion protein-1 in smooth muscle cells. Am J Pathol. 1999;155:1953–65. 10595925 10.1016/S0002-9440(10)65514-9 PMC1866916

[39] Moldes M, Fève B, Pairault J. Molecular cloning of a major mRNA species in murine 3T3 adipocyte lineage. differentiation-dependent expression, regulation, and identification as semicarbazide-sensitive amine oxidase. J Biol Chem. 1999;274:9515–23. 10092636 10.1074/jbc.274.14.9515

[40] Salmi M, Jalkanen S. VAP-1: an adhesin and an enzyme. Trends Immunol. 2001;22:211–6. 11274927 10.1016/s1471-4906(01)01870-1

[41] Airas L, Mikkola J, Vainio JM, Elovaara I, Smith DJ. Elevated serum soluble vascular adhesion protein-1 (VAP-1) in patients with active relapsing remitting multiple sclerosis. J Neuroimmunol. 2006;177:132–5. 16806498 10.1016/j.jneuroim.2006.05.014

[42] O’Rourke AM, Wang EY, Salter-Cid L, Huang L, Miller A, Podar E, et al. Benefit of inhibiting SSAO in relapsing experimental autoimmune encephalomyelitis. J Neural Transm (Vienna). 2007;114:845–9. 17393060 10.1007/s00702-007-0699-3

[43] Weston CJ, Shepherd EL, Claridge LC, Rantakari P, Curbishley SM, Tomlinson JW, et al. Vascular adhesion protein-1 promotes liver inflammation and drives hepatic fibrosis. J Clin Invest. 2015;125:501–20. 25562318 10.1172/JCI73722 PMC4319424

[44] Viitanen R, Virtanen H, Liljenbäck H, Moisio O, Li X-G, Nicolini V, et al. [^68^Ga]Ga-DOTA-Siglec-9 detects pharmacodynamic changes of FAP-targeted IL2 variant immunotherapy in B16-FAP melanoma mice. Front Immunol. 2022;13:901693. 35874707 10.3389/fimmu.2022.901693 PMC9298541

[45] Hena KM. Sarcoidosis epidemiology: race matters. Front Immunol. 2020;11:537382. 33042137 10.3389/fimmu.2020.537382 PMC7522309

