# Supplementary material for: Proof-of-concept PET imaging of pulmonary sarcoidosis using VAP-1-targeted radiotracer [68Ga]Ga-DOTA-Siglec-9
Source: Respir Res. 2025 Dec 19;27:124. doi: 10.1186/s12931-025-03455-8 (PMC12977699; doi:10.1186/s12931-025-03455-8)
Supplement: Supplementary file 1 — Supplementary Material 1. [file 12931_2025_3455_MOESM1_ESM.pdf]

Supplementary information

Proof-of-concept PET imaging of pulmonary sarcoidosis using VAP-1-targeted radiotracer [^{68}Ga]Ga-DOTA-Siglec-9

Prince Dadson[1,2,3], Heli Ylä-Outinen[4], Kari Kalliokoski[1,2], Terhi Tuokkola[1,2], Simona Malaspina[1,2], Mikko Koivumäki[1,2], Riikka Viitanen[1], Noora Rajala[1], Maria Silvoniemi[4], Tuula Tolvanen[2,5], Pirjo Nuutila[1,2,6], Sirpa Jalkanen[6,7], Antti Saraste[1,2,6,8], Tarja Saaresranta[4,9], Pekka Taimen[6,10], and Anne Roivainen[1,2,6*]

[1]Turku PET Centre, University of Turku, Turku, Finland; [2]Turku PET Centre, Turku University Hospital, Turku, Finland; [3]Turku PET Centre, Åbo Akademi University, Turku, Finland; [4]Department of Pulmonary Diseases, Turku University Hospital, Turku, Finland; [5]Department of Medical Physics, Division of Medical Imaging, Turku University Hospital, Turku, Finland; [6]InFLAMES Research Flagship Center, University of Turku, Turku, Finland; [7]MediCity Research Laboratory, University of Turku, Turku, Finland; [8]Heart Center, Turku University Hospital and University of Turku, Turku, Finland; [9]Department of Pulmonary Diseases and Clinical Allergology, University of Turku, Turku, Finland; [10]Institute of Biomedicine, University of Turku and Department of Pathology, Turku University Hospital, Turku, Finland

***Correspondence**: Prof. Anne Roivainen, PhD, Turku PET Centre, Kiinamyllynkatu 4-8, FI-20521 Turku, Finland. E-mail anne.roivainen@utu.fi

Supplementary materials and methods

Radiopharmaceuticals

All PET radiopharmaceuticals synthesized at the Radiopharmaceutical Laboratory of Turku PET Centre under Turku University Hospital Pharmacy control, and were compliant with Good Manufacturing Practice (GMP) guidelines. Reagents purchased from commercial suppliers were either synthesis grade or analytical grade.

Synthesis of 2-deoxy-2-[^{18}F]-fluoro-*D*-glucose ([^{18}F]FDG)

[^{18}F]FDG was synthesized using a FASTlab synthesizer (GE Healthcare, Waukesha, WI, USA) and an FDG-phosphate cassette, as described by Long et al. [1]. Fluoride-18 was generated by irradiating oxygen-18 enriched water (GMP-grade, 98%, Rotem Industries Ltd, Medical Imaging, Dimona, Israel) using 17 MeV protons in a CC-18/9 cyclotron (Efremov Institute of Electrophysical Apparatuses, St Petersburg, Russia) with a beam current of 40 µA. The synthesis resulted in a molar activity >100 GBq/µmol and a radiochemical purity >95%, as confirmed by thin-layer chromatography and high-performance liquid chromatography (HPLC). Total synthesis time was 45 min.

Synthesis of Gallium-68-labeled 1,4,7,0-tetraazacyclododecane-1,4,7,10-tetraacetic acid conjugated sialic acid-binding immunoglobulin-like lectin 9 motif containing peptide ([^{68}Ga]Ga-DOTA-Siglec-9)

The GMP grade precursor DOTA-Siglec-9 was obtained from ABX Advanced Biomedical Compounds GmbH (Radeberg, Germany) as a custom synthesis. Radiosynthesis was performed as previously described using a fully automated synthesis device (Modular Lab PharmTracer; Eckert & Ziegler, Berlin, Germany); the process complied with all GMP requirements [2]. ^{68}Ga was obtained from a ^{68}Ge/^{68}Ga generator (GalliaPharm, 1.85 GBq; Eckert and Ziegler, Berlin, Germany) by eluting

the generator with 6 mL of 0.1 M HCl and passing the eluate through a Strata-XC cation exchange cartridge (Phenomenex Inc., Torrance, CA, USA). Bound $^{68}GaCl_3$ was eluted with acidified acetone (0.8 mL, containing 3.25% water and 0.02 M HCl) into a reaction vial preloaded with a mixture of DOTA-Siglec-9 (80 µL, 500 µg/mL), sodium acetate buffer (2.0 mL, 0.2 M, pH 4.0), and absolute ethanol (0.2 mL). The reaction mixture was incubated at 65°C for 6 min and then diluted with saline (5 mL, 0.9 mg/mL). The crude product was purified by being loaded onto a C18 cartridge (SepPak Light C18; Waters, Milford, MA, USA), which was then washed with saline (10 mL). [^{68}Ga]Ga-DOTA-Siglec-9 was eluted with ethanol (1.3 mL, 70% [*v*/*v*]) through a nonpyrogenic 0.22-µm filter into the sterile final-product vial. The product was formulated in physiologic saline, and the final volume of the end product was 10 mL. Total synthesis time was 25 min.

The radiochemical purity of the product was evaluated by radio-HPLC (LC-20A Prominence HPLC System [Shimadzu, Kyoto, Japan] and an online radioactivity detector Flow-Count [Bioscan Inc., Washington, DC, USA]) fitted with an analytic Kinetex C18 column (2.6 µm, 100 Å, 75 × 4.6 mm; Phenomenex, Torrance, CA, USA) running at a flow rate of 1.0 mL/min with a gradient of 0.16% trifluoroacetic acid in water (A) and 0.16% trifluoroacetic acid in acetonitrile (B) (gradient switched from 18% B to 50% B over 12 min).

PET/CT scanners and radiation exposure

Imaging was conducted using the Discovery 690 or Discovery MI (PET/CT (General Electric Medical Systems, Milwaukee, WI, USA) systems. The Discovery 690 hybrid PET/CT system, equipped with a lutetium-yttrium oxyorthosilicate block detector and a 64-slice CT scanner, provides high spatial resolution and sensitivity [3]. The 4-ring digital Discovery MI showed enhanced image quality and sensitivity, with superior spatial resolution, increased sensitivity, and a higher noise equivalent count rate peak [4]. The CT slice thickness for the Discovery 690 was 3.27 mm, with an average radiation exposure of 4.5 mSv from a whole-body CT scan for attenuation correction (CTAC). For the

Discovery MI, the CT slice thickness was 2.79 mm, and the average radiation exposure from CTAC was 8.2 mSv.

The effective radiation dose for a 140 MBq injection of [^{68}Ga]Ga-DOTA-Siglec-9 was estimated to be 3.08 mSv (0.022 mSv/MBq) [5], and that for a dose of 200 MBq of [^{18}F]FDG was estimated to be 3.8 mSv (0.019 mSv/MBq) [6]. An independent [^{18}F]FDG PET/CT study using the Discovery 690 showed that the total radiation dose was 8.0 mSv (4.5 mSv from CTAC plus 3.8 mSv from [^{18}F]FDG), corresponding to the average annual radiation dose in Finland received over a period of 1 year and 4 months. Similarly, the [^{68}Ga]Ga-DOTA-Siglec-9 PET/CT study with the Discovery 690 revealed that the total radiation dose was 7.6 mSv (4.5 mSv from CTAC plus 3.08 mSv from [^{68}Ga]Ga-DOTA-Siglec-9), equating to the average annual radiation dose in Finland received over 1 year and 3 months. For the Discovery MI, the total radiation dose in an [^{18}F]FDG PET/CT study was 12.0 mSv (8.2 mSv from CTAC plus 3.8 mSv from [^{18}F]FDG), representing the average annual radiation dose in Finland received over 2 years. In the [^{68}Ga]Ga-DOTA-Siglec-9 PET/CT study with the Discovery MI, the total radiation dose was 11.3 mSv (8.2 mSv_from CTAC plus 3.08 mSv from [^{68}Ga]Ga-DOTA-Siglec-9), equivalent to the average annual radiation dose in Finland received over 1 year and 10 months.

PET/CT imaging of a lung cancer patient was performed using Vision Quadra (Siemens Healthineers).

References

1. Long JZ, Jacobson MS, Hung JC. Comparison of FASTlab ^{18}F-FDG production using phosphate and citrate buffer cassettes. J Nucl Med Technol. 2013;41:32–4. https://doi.org/0.2967/jnmt.112.112649
2. Käkelä M, Luoto P, Viljanen T, Virtanen H, Liljenbäck H, Jalkanen S, et al. Adventures in radiosynthesis of clinical grade [^{68}Ga]Ga-DOTA-Siglec-9. RSC Adv. 2018;8:8051–6. https://doi.org/10.1039/c7ra12423f

3. Bettinardi V, Presotto L, Rapisarda E, Picchio M, Gianolli L, Gilardi MC. Physical performance of the new hybrid PET∕CT Discovery-690. Med Phys. 2011;38:5394–411. https://doi.org/10.1118/1.3635220

4. Chicheportiche A, Marciano R, Orevi M. Comparison of NEMA characterizations for Discovery MI and Discovery MI-DR TOF PET/CT systems at different sites and with other commercial PET/CT systems. EJNMMI Phys. 2020;7:4. https://doi.org/10.1186/s40658-020-0271-x

5. Viitanen R, Moisio O, Lankinen P, Li X-G, Koivumäki M, Suilamo S, et al. First-in-humans study of ^{68}Ga-DOTA-Siglec-9, a PET ligand targeting vascular adhesion protein 1. J Nucl Med. 2021;62:577–83. https://doi.org/10.2967/jnumed.120.250696

6. Addendum 1 to ICRP Publication 128: Radiation dose to patients from radiopharmaceuticals: a compendium of current information related to frequently used substances [Ann. ICRP 44(2S), 2015]. Ann ICRP. 2020;146645320936035. https://doi.org/10.1177/0146645320936035

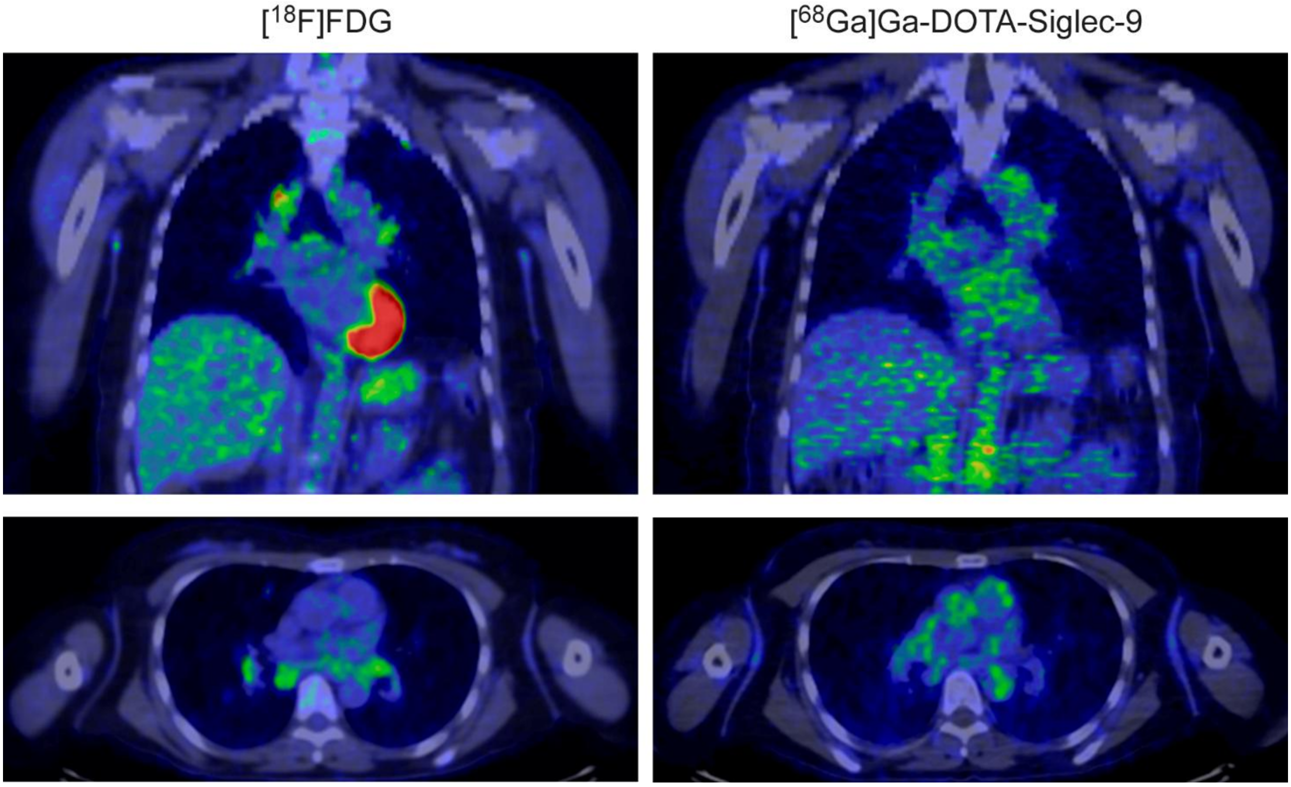

Supplementary Fig. 1 Fused [^{18}F]FDG and [^{68}Ga]Ga-DOTA-Siglec-9 PET/CT images of Case P001 (female, 48 years). [^{18}F]FDG images showed prominent abnormal activity in the hilar lymph nodes, with a total area SUV_{max} of 5.6. No other uptake suggestive of sarcoidosis was observed on the [^{18}F]FDG PET/CT. In the [^{68}Ga]Ga-DOTA-Siglec-9 PET/CT images, activity was observed in the hilar lymph nodes, although it was less extensive and less distinct than the findings in the [^{18}F]FDG scan.

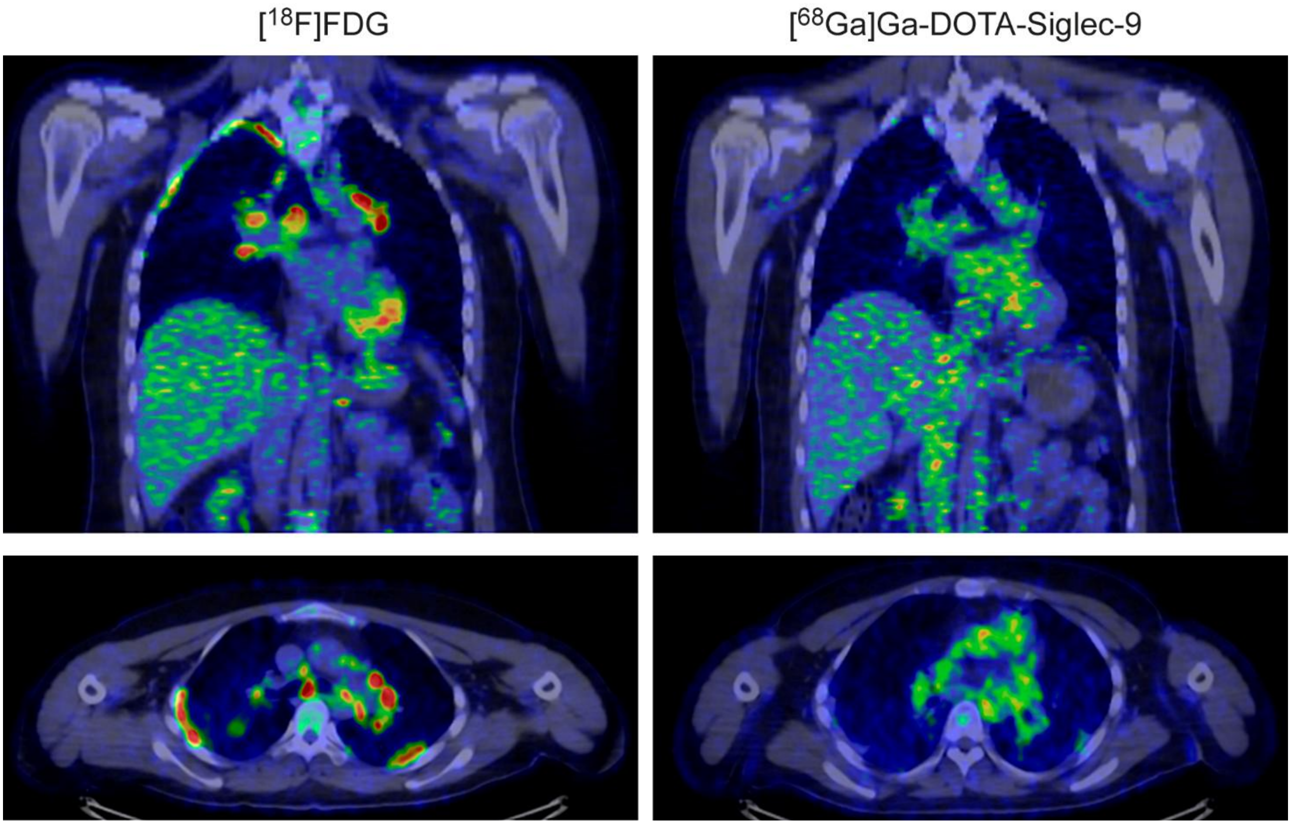

Supplementary Fig. 2 Fused [^{18}F]FDG and [^{68}Ga]Ga-DOTA-Siglec-9 PET/CT images of Case P002 (male, 28 years). In the [^{18}F]FDG PET images of the neck, caudally to the thyroid gland on both sides and predominantly in the left supraclavicular region, a few active lymph nodes of normal size or slightly enlarged were noted, with SUV_{max} values ranging from 3.1–6.6. Abnormal activity was observed in mediastinal (SUV_{max} 6.0) and hilar (SUV_{max} 7.1) regions. Lung parenchymal changes were observed apically, with SUV_{max} values of 2.2 on the right and 2.0 on the left. Additionally, peripleural parenchymal consolidations were noted (SUV_{max} 5.8–6.1 on the right and 6.5 on the left). One active lymph node was also detected below the gastroesophageal junction, measuring 9 mm and demonstrating a SUV_{max} of 7.1. No other uptake suggestive of sarcoidosis was found on the [^{18}F]FDG PET/CT. Hilar activity was visible in the [^{68}Ga]Ga-DOTA-Siglec-9 PET/CT images. Some activity was also observed in the peripleural lung areas, though less extensive than on the [^{18}F]FDG scan.

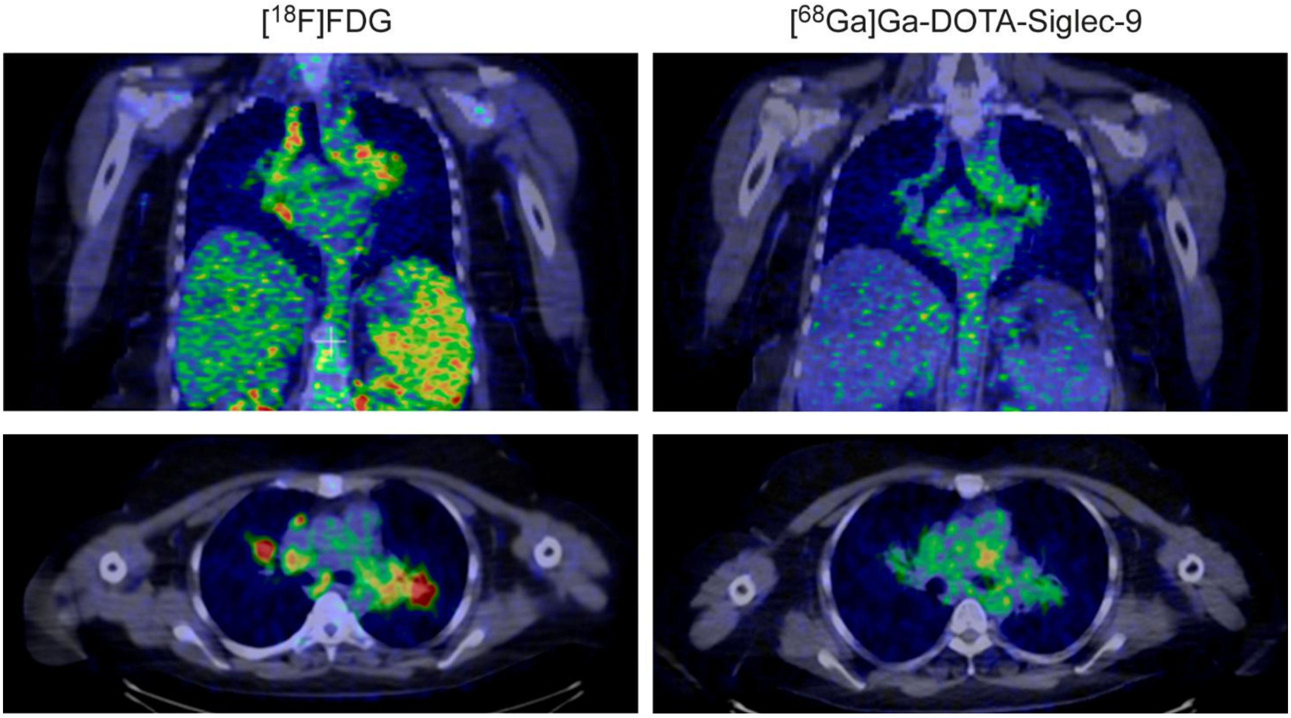

Supplementary Fig. 3 Fused [^{18}F]FDG and [^{68}Ga]Ga-DOTA-Siglec-9 PET/CT images of Case P003 (female, 58 years). In the [^{18}F]FDG PET/CT images, a 14-mm lymph node with a SUV_{max} of 4.9 was noted in the left supraclavicular region. Numerous large and active lymph nodes were present in the mediastinum. Specifically, in the right upper mediastinum, the lymph node area exhibited a SUV_{max} of 10.5. Parenchymal changes in the left upper lobe were also observed, with a SUV_{max} of 6.4. In the liver hilum area, a 7 × 4 cm lymph node mass with a SUV_{max} of 10.7 was noted. Additionally, several large para-aortic lymph nodes were present in the upper abdomen, with an area SUV_{max} of 16.4. In the mesentery on the right side of the midline, a few large and active lymph nodes with an area SUV_{max} of 12.1 were noted. In the left groin, a 16-mm lymph node with a SUV_{max} of 13.3 was observed, and in the right groin, an 11-mm lymph node with a SUV_{max} of 13.3 was detected. Activation patterns in the [^{68}Ga]Ga-DOTA-Siglec-9 PET/CT images were similar to those seen on the [^{18}F]FDG images, although the areas were distinctly less active on the [^{68}Ga]Ga-DOTA-Siglec-9 images.

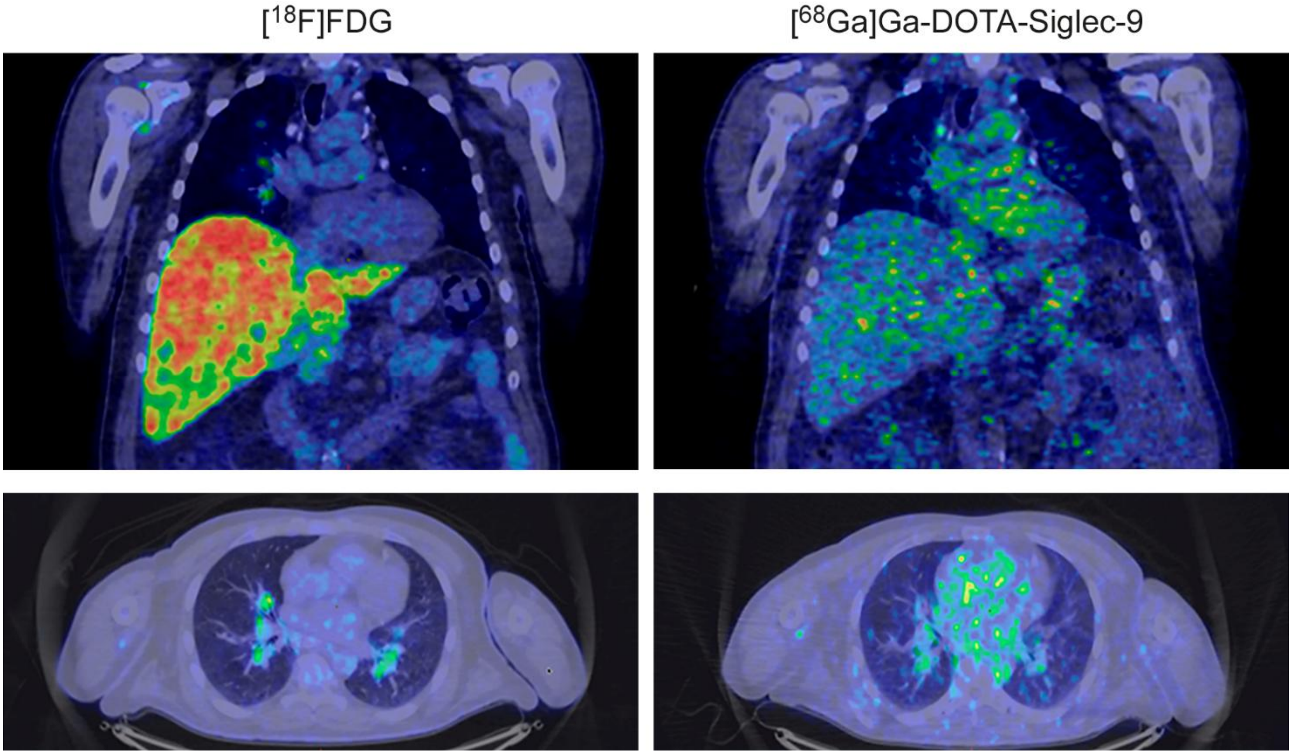

Supplementary Fig. 4 Fused [^{18}F]FDG and [^{68}Ga]Ga-DOTA-Siglec-9 PET/CT images of Case P004 (male, 50 years). [^{18}F]FDG images show increased tracer accumulation in the bilateral peribronchovascular regions of the lung hilum, with SUV_{max} values of 5.2 on the left and 4.2 on the right. The liver exhibited patchy abnormal activity throughout, with a SUV_{max} of 9.3. In the upper abdomen, around the head of the pancreas and para-aortically, a few slightly active lymph nodes were noted with a SUV_{max} of 4.8. Further down, para-aortically and adjacent to the external iliac arteries on both sides, moderately active lymph nodes were observed (SUV_{max} of 5.0). Small patchy accumulations were seen in the bones, most prominently in the diaphyseal regions of the femurs, the acetabulum (bilaterally), the sacrum, and the left clavicle (medially). The most active skeletal accumulations, with a SUV_{max} of 5.5, were observed in the right sacrum and right femur. No lytic bone destruction was visible. In the [^{68}Ga]Ga-DOTA-Siglec-9 PET/CT images, tracer accumulation was observed in the peribronchovascular regions of the lung hilum, similar to the [^{18}F]FDG findings; however, the extent and intensity of activity were lower than observed on the [^{18}F]FDG scan.

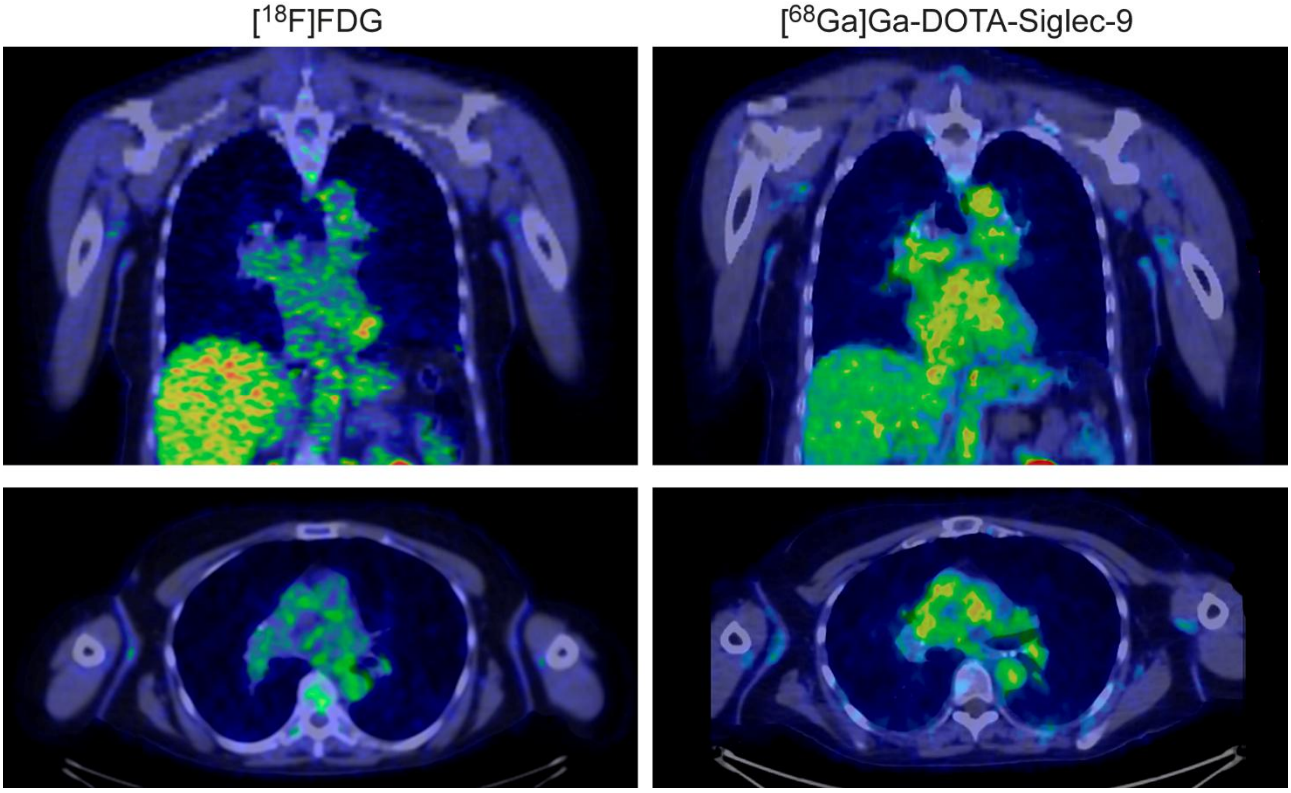

Supplementary Fig. 5 Fused [^{18}F]FDG and [^{68}Ga]Ga-DOTA-Siglec-9 PET/CT images of Case P005 (female, 62 years). In the [^{18}F]FDG PET/CT images of the left lower lung lobe, honeycomb-like shadowing was observed. Some activity (SUV_{max} 4.0) was present, suggesting an inflammatory process, although the pattern was not characteristic of sarcoidosis. Para-aortically, several active but normal-sized lymph nodes were noted in the upper abdomen, with a SUV_{max} values ranging from 9.6 to 19.1. In the [^{68}Ga]Ga-DOTA-Siglec-9 PET/CT images, faint activity was seen in the lungs and para-aortic lymph nodes, lower than the activity observed on the [^{18}F]FDG images.

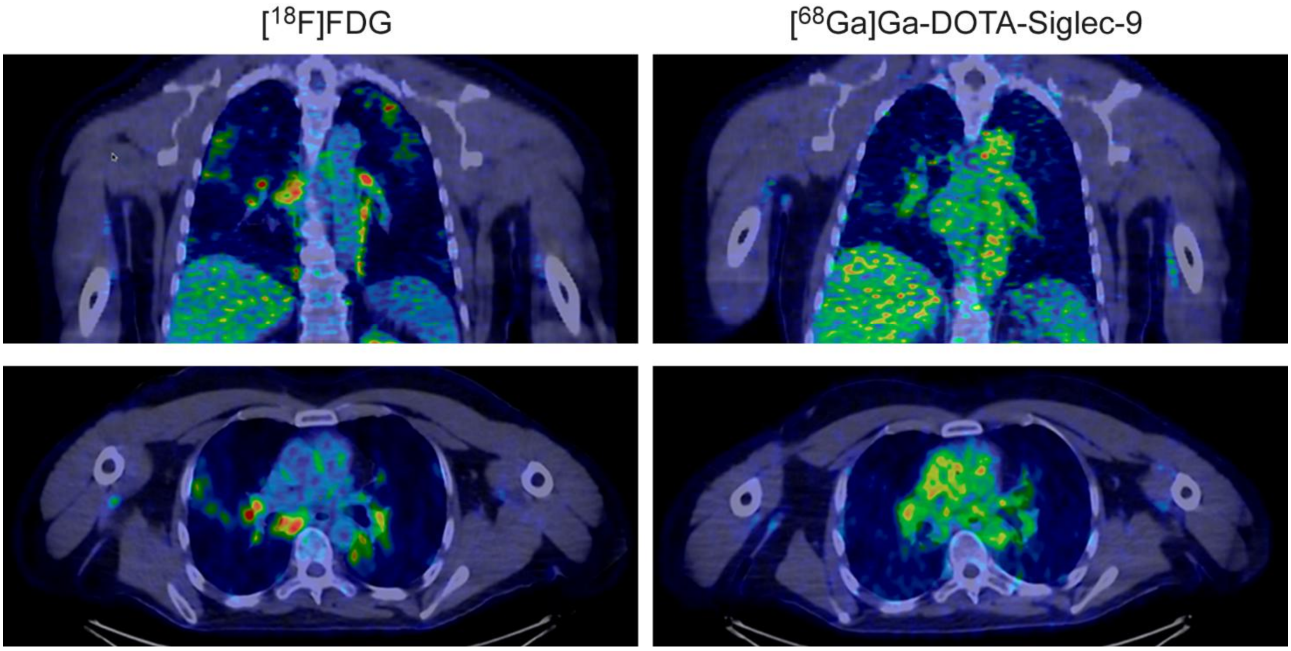

Supplementary Fig. 6 Fused [^{18}F]FDG and [^{68}Ga]Ga-DOTA-Siglec-9 PET/CT images of Case P006 (male, 59 years). [^{18}F]FDG PET/CT images show several active lymph nodes in the parathyroid region, mediastinum, and hilum, varying in size. The SUVs for these regions were as follows: parathyroid region, SUV_{max} 5.4; mediastinum, SUV_{max} 12.0; and hilum, SUV_{max} 10.2. Active sarcoidosis-related changes were observed in all lung lobes, with SUVs ranging from 5.3 to 7.0. In the right crus of the diaphragm, an 11-mm lymph node was noted with a SUV_{max} of 9.0. In the upper abdomen, the liver, biliary system, pancreas, spleen, adrenal glands, and kidneys appeared normal on low-dose CT. In the upper abdomen, anterior to the aorta, several lymph nodes measuring 12–13 mm were identified, with a SUV_{max} of 6.2. At the level of the renal arteries, a few large para-aortic lymph nodes were observed, with an area SUV_{max} of 8.2. Para-iliac lymph nodes on both sides, mostly of normal size, were present, with SUVs of 6.7. In the groin, both normal-sized and enlarged lymph nodes were noted, with an area SUV_{max} of 4.7 on the left and 6.6 on the right. In the [^{68}Ga]Ga-DOTA-Siglec-9 PET/CT images, there was notable activity in the lungs and mediastinal lymph nodes, consistent with the active changes observed on the [^{18}F]FDG scan. The para-aortic and para-iliac lymph nodes displayed moderate uptake, aligning with the findings from the [^{18}F]FDG scan.

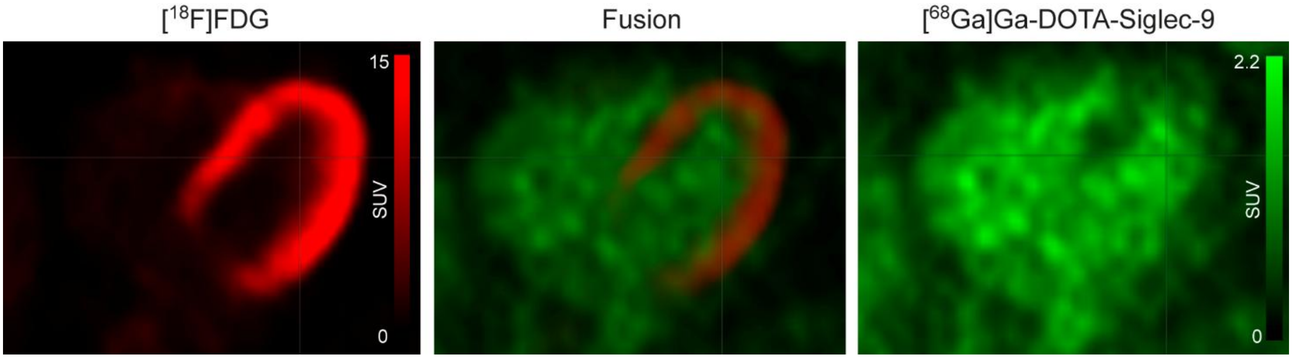

Supplementary Fig. 7 Horizontal fused [^{68}Ga]Ga-DOTA-Siglec-9 and [^{18}F]FDG images centered at the left ventricle show diffuse physiological uptake of [^{18}F[FDG (red) in the myocardium, whereas [^{68}Ga]Ga-DOTA-Siglec-9 signal (green) is lower than in the blood throughout the left ventricular myocardium.

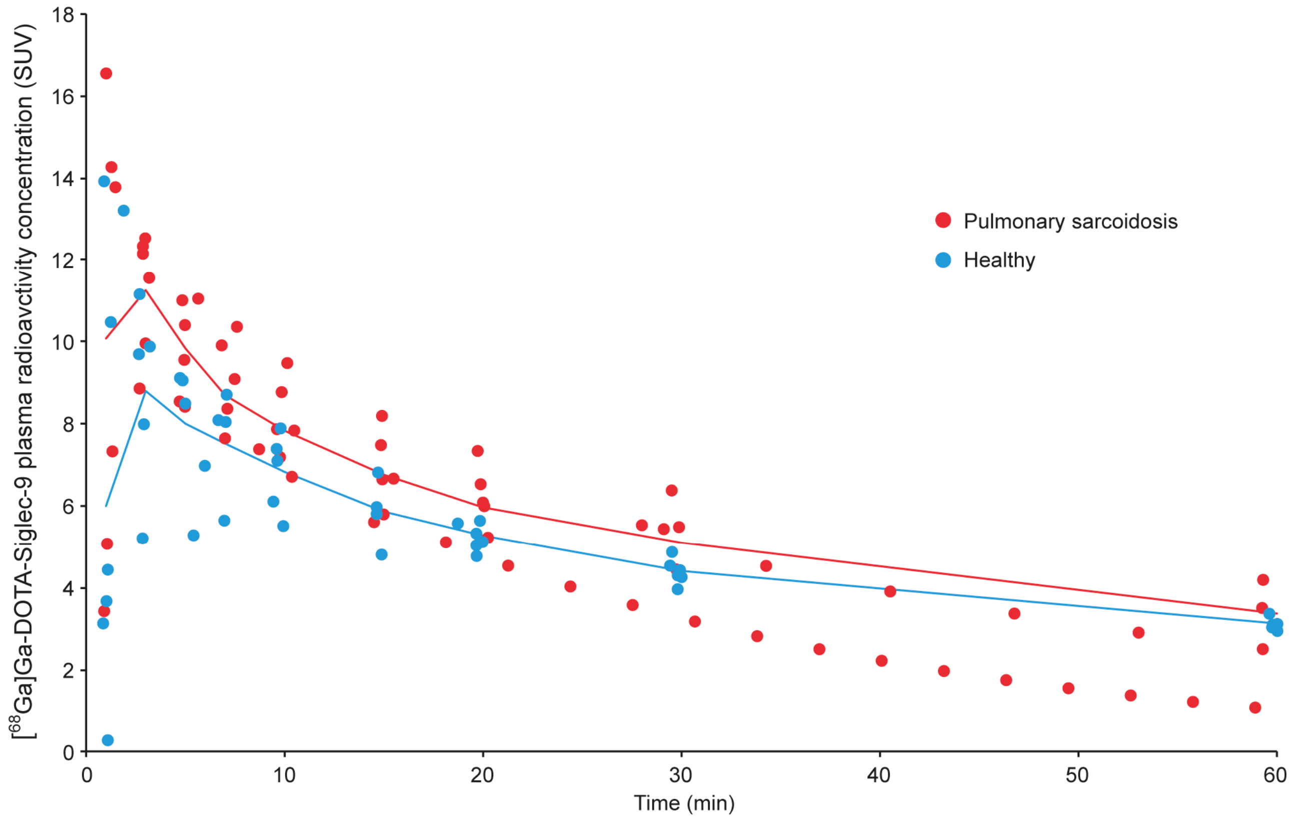

Supplementary Fig. 8 Plasma concentration of radioactivity as a function of time after intravenous injection of [^{68}Ga]Ga-DOTA-Siglec-9. Red dots denote patients with pulmonary sarcoidosis and blue dots denote healthy subjects. Red and blue lines represent the mean values for sarcoidosis patients and healthy subjects, respectively.

Supplementary Table 1 Comprehensive clinical data from the patients with sarcoidosis

Patient (gender)	P001 (female)	P002 (male)	P003 (female)	P004 (male)	P005 (female)	P006 (male)
**Histological confirmation**	FNA 4R, 7	FNA 12R, 7<br>Bronchial biopsy	Mediastinoscopy 4R	Liver biopsy	Bronchial biopsy	FNA 7, 4R
**Other diagnoses**	Lymphocytic colitis, Vertebrobasilar TIA, Tendency to iron deficiency, Hypercholesterolemia	Attention Deficit Disorder	Bipolar disorder, Prediabetes, Hypercholesterolemia	Hypertension, Hypercholesterolemia, Gout, Sleep apnea (CPAP), T2DM, BPH,<br>CHD with a history of MI, Asymptomatic brain infarct, Cardiac insufficiency, History of alcoholic pancreatitis, Sarcoidosis-related tubulointerstitial nephritis, cirrhosis (Child-Pug A) and renal insufficiency	Hypertension, Hypercholesterolemia	Allergic rhinoconjunctivitis (pollens, animals)
**Medication at diagnosis**	Simvastatine, Citalopram, Budesonid (ileal release formulation; one-week pause before the [^{68}Ga]Ga-DOTA-Siglec-9 PET scans)	No medication	Aspirin	Amlodipine, Bisoprolol, Ramipril, Prazosin. Insulin Glargine, Semaglutide, Aspirin, Furosemide, Atorvastatin, Tamsulosin, Allopurinol	Bisoprolol, Atorvastatin, Calsium, Vitamin-D	As needed antihistamines<br>-
**Symptoms at diagnosis**	Asymptomatic, adenopathy observed on CT scan (TIA)	Dyspnea, cough, increased tiredness	Joint swelling and pain, chest pain (Tietze syndrome),<br>fatigue and night sweats, morning stiffness of joints on hands and knees	Acute renal insufficiency and hypercalcemia treated at ICU, minor symptoms of dactylitis and pain	Dry cough, occasional chest pain	Tiredness, dyspnea, chest pain attacks
**Smoking history**	Non-smoker	Non-smoker	Non-smoker	Non-smoker	Non-smoker	Non-smoker
**HRCT/PET-CT findings**	Symmetric mediastinal lymphadenopathy, pleural nodularity, and peribronchovascular nodularity	Symmetric mediastinal lymphadenopathy, parenchymal consolidations, parenchymal nodules, and interlobular septal thickening	Increased number of mediastinal lymph nodes, peribronchovascular infiltrates, crazy paving, and ground-glass opacities	Mediastinal lymphadenopathy with calcifications, and peribronchovascular infiltrates	Mediastinal lymphadenopathy, perilymphatic parenchymal nodules, and ground-glass opacities	Mediastinal lymphadenopathy, and perilymphatic and perifissural parenchymal nodules

**Spirometry**	Normal<br>FEV1: 2.8 L (93% of predicted)<br>FVC: 3.59 L (94% of predicted)<br>FEV1/FVC: 0.78 (99% of predicted)	Moderate restriction<br>FEV1: 4.05 L (74% of predicted), Z-score -2.97 (8),<br>FVC: 5.21 L (76% of predicted), Z-score -3.07<br>FEV1/FVC: 0.78 (98% of predicted), Z-score -0.21	Normal<br>FEV1: 2.79 L (81% of predicted), Z-score -1.26<br>FVC: 3.85 L (81% of predicted), Z-score -1.26<br>FEV1/FVC: 0.75 (101% of predicted), Z-score 0.14	Normal<br>FEV1: 3.58 L (81% of predicted), Z-score -1.49<br>FVC: 4.38 L (79% of predicted), Z-score -1.74<br>FEV1/FVC: 0.79 (103% of predicted), Z-score 0.42	Normal<br>FEV1: 2.21 L (86% of predicted), Z-score -1.09<br>FVC: 3.10 L (92% of predicted), Z-score -0.61<br>FEV1/FVC: 0.71 (94% of predicted), Z-score -0.92	Moderate restriction<br>FEV1: 2.2 L (65% of predicted), Z-score -2.1<br>FVC: 3.3 L (73% of predicted), Z-score -1.7<br>FEV1/FVC: 0.68 (89% of predicted), Z-score -1.5
**Diffusion capacity**	Not performed	Not performed	Slightly decreased<br>DLCOc: 69%<br>DLCOc/VA: 80%	Normal<br>DLCOc: 82%<br>DLCO/VA: 104%	Not performed	Normal<br>DLCOc: 82%<br>DLCOc/VA: 105%

Abbreviations: 4R (right lower paratracheal lymph node), 7 (subcarinal lymph node), 12R (right hilar lymph node), designation of lymph node stations in the mediastinum; BPH, benign prostatic hyperplasia; CHD, coronary heart disease; CPAP, continuous positive airway pressure device; CT, computed tomography; DLCOc, diffusing capacity of the lungs for carbon monoxide, corrected for hemoglobin; DLCO/VA, diffusing capacity of the lungs for carbon monoxide adjusted for alveolar volume; T2DM, type 2 diabetes mellitus; FEV1, forced expiratory volume in one second; FNA, fine-needle aspiration; FVC, forced vital capacity; HRCT, high-resolution computed tomography; MI, myocardial infarction; PET-CT, positron emission tomography-computed tomography; TIA, transient ischemic attack.

Supplementary Table 2 Hematology, serology, and clinical chemistry data

Parameter	Pulmonary sarcoidosis (n = 6)	Healthy controls (n = 6)	P value	Reference value
White blood cell count (10^9/L)	5.9 ± 2.3	6.1 ± 2.4	0.438	3.4–8.2
Red blood cell count (10^{12}/L)	4.6 ± 0.3	5.0 ± 0.5	0.067	3.9–5.2[a]; 4.3–5.7[b]
Hemoglobin (g/L)	134 ± 8.9	147 ± 6.6	0.014	117–155[a]; 134–167[b]
Hematocrit	0.41 ± 0.03	0.43 ± 0.02	0.073	0.35–0.46[a]; 0.39–0.50[b]
Mean corpuscular volume (fL)	88.9 ± 3.8	87.4 ± 3.0	0.254	82–98
Mean corpuscular hemoglobin (pg)	29.3 ± 1.8	30.0 ± 1.2	0.246	27–33
Platelet count (10^9/L)	246.5 ± 57.6	202 ± 49.2	0.104	150–360
Neutrophil count (10^9/L)	3.7 ± 1.6	3.5 ± 2.3	0.426	1.3–3.6[a]; 1.5–6.7[b]
Lymphocyte count (10^9/L)	1.3 ± 0.5	1.8 ± 0.3	0.036	0.2–0.8[a]; 1.3–3.6[b]
Monocyte count (10^9/L)	0.6 ± 0.2	0.6 ± 0.2	0.435	0.03–0.44[a]; 0.2–0.8[b]
Eosinophil count (10^9/L)	0.25 ± 0.13	0.18 ± 0.11	0.183	0–0.1[a]; 0.03–0.44[b]
Basophil count (10^9/L)	0.05 ± 0.04	0.05 ± 0.02	0.472	0.0–0.1
Absolute neutrophils (%)	62.2 ± 3.3	53.8 ± 11.9	0.058	41–81
Absolute lymphocytes (%)	21.5 ± 3.5	32.4 ± 11.1	0.023	20–45
Absolute monocytes (%)	10.7 ± 2.0	9.8 ± 1.9	0.241	1–11
Absolute eosinophils (%)	4.5 ± 2.1	3.6 ± 2.7	0.273	1–5
Absolute basophils (%)	1.0 ± 0.63	1.0 ± 0.7	0.500	0–1
Erythrocyte sedimentation rate (mm/h)	12.7 ± 16.0	3.2 ± 1.6	0.112	<30[a]; <15[b]
Procalcitonin (μg/L)	0.07 ± 0.07	ND	ND	<0.05
C-reactive protein (mg/L)	3.5 ± 3.7	1.0 ± 0.0	0.083	<10
Potassium (mmol/L)	3.8 ± 0.3	3.9 ± 0.2	0.218	3.3–4.8
Sodium (mmol/L)	139 ± 2.9	142 ± 1.1	0.058	137–144
Creatinine (μmol/L)	110.5 ± 69.1	82 ± 12.3	0.198	50–90[a]; 60–100[b]
Alkaline phosphatase (U/L)	154 ± 213.6	65 ± 11.6	0.189	35–105
Alanine aminotransferase (U/L)	38.8 ± 37.2	36.2 ± 17.0	0.444	<35[a]; <50[b]
Rheumatoid factor (IU/mL)	30.3 ± 41.6	ND	ND	<7[a]; <14[b]
Citrullinated peptide antibody (U/mL)	7.5 ± 1.2	ND	ND	<7

Values are expressed as the mean ± SD and range. P values are from the independent samples t-test. ND, not determined. [a]For female. [b]For male.

Supplementary Table 3 Comparison of tissue-specific SUV_{max} values for [^{68}Ga]Ga-DOTA-Siglec-9 and [^{18}F]FDG in patients with sarcoidosis

Tissue	[^{68}Ga]Ga-DOTA-Siglec-9 (*n* = 6)	[^{18}F]FDG (*n* = 6)	*P* value
Blood, heart left ventricular cavity	2.91 ± 0.86	1.73 ± 0.53	0.009
Bone, cortical	0.49 ± 0.08	0.63 ± 0.12	0.027
Bone marrow	0.58 ± 0.13	1.30 ± 0.27	0.001
Kidneys	15.56 ± 11.76	14.16 ± 11.43	0.832
Liver	2.61 ± 0.94	4.00 ± 0.99	0.076
Lung parenchyma	2.01 ± 0.44	1.01 ± 0.24	0.009
Mediastinal lymph node	2.49 ± 0.53	4.22 ± 2.43	0.087
Muscle, triceps brachii	0.64 ± 0.21	0.59 ± 0.12	0.712
Myocardium	1.54 ± 0.38	4.63 ± 4.44	0.168
Pancreas	1.83 ± 0.56	1.86 ± 0.65	0.870
Parotid gland	1.09 ± 0.42	1.49 ± 0.44	0.203
Spleen	2.18 ± 0.62	2.92 ± 1.17	0.181
Salivary gland, submandibular	1.16 ± 0.48	2.08 ± 0.83	0.059
Thymus	1.10 ± 0.40	0.89 ± 0.45	0.375
Urinary bladder content	127.26 ± 140.92	34.02 ± 35.74	0.083

The results are expressed as the maximum standardized uptake values (SUV_{max}, mean ± SD). *P* values are from the independent-sample *t*-test.

Supplementary Table 4 Tissue-to-blood ratio of [^{68}Ga]Ga-DOTA-Siglec-9 uptake

	Pulmonary sarcoidosis (n = 6)	Healthy controls (n = 6)	P value
Bone, cortical	0.34 ± 0.09	0.17 ± 0.09	0.001
Bone marrow	0.40 ± 0.12	0.22 ± 0.15	0.007
Kidneys	10.61 ± 7.63	6.68 ± 2.08	0.293
Liver	6.64 ± 2.00	7.91 ± 3.11	0.305
Lung parenchyma	1.40 ± 0.45	0.46 ± 0.14	0.003
Mediastinal lymph node	1.73 ± 0.56	0.64 ± 0.13	0.003
Muscle, triceps brachii	0.43 ± 0.14	0.39 ± 0.17	0.621
Myocardium	1.04 ± 0.22	0.83 ± 0.27	0.044
Pancreas	1.27 ± 0.46	0.82 ± 0.26	0.096
Parotid gland	0.75 ± 0.32	0.64 ± 0.22	0.444
Spleen	1.52 ± 0.55	0.88 ± 0.19	0.047
Salivary gland, submandibular	0.82 ± 0.41	0.70 ± 0.20	0.560
Thymus	0.75 ± 0.25	0.80 ± 0.33	0.767

The results are expressed as mean ± SD. Tissue-to-blood ratio = $SUV_{max\_tissue}/SUV_{mean\_blood}$. P values are from the independent-samples t-test.

Supplementary Table 5 Tissue-to-muscle ratio of [^{68}Ga]Ga-DOTA-Siglec-9 uptake

	Pulmonary sarcoidosis (n = 6)	Healthy controls (n = 6)	P value
Bone, cortical	1.67 ± 0.46	0.77 ± 0.32	0.002
Bone marrow	1.97 ± 0.59	1.04 ± 0.63	0.006
Kidneys	53.75 ± 39.35	31.16 ± 8.07	0.216
Liver	1.36 ± 0.40	1.70 ± 0.70	0.157
Lung parenchyma	4.48 ± 2.15	2.11 ± 0.50	0.064
Mediastinal lymph node	5.81 ± 2.77	3.02 ± 0.70	0.055
Myocardium	5.09 ± 1.02	3.95 ± 1.61	0.121
Pancreas	6.33 ± 2.48	4.06 ± 1.98	0.141
Parotid gland	3.67 ± 1.51	3.05 ± 1.13	0.457
Spleen	7.48 ± 2.80	4.16 ± 1.19	0.069
Salivary gland, submandibular	4.04 ± 1.99	3.36 ± 1.38	0.565
Thymus	3.63 ± 1.10	3.75 ± 1.46	0.899

The results are expressed as mean ± SD. Tissue-to-muscle ratio = $SUV_{max\_tissue}/SUV_{mean\_muscle}$. P values are from the independent-samples t-test.
